# Advancements in the Additive Manufacturing of Magnesium and Aluminum Alloys through Laser-Based Approach

**DOI:** 10.3390/ma15228122

**Published:** 2022-11-16

**Authors:** Sachin Kumar Sharma, Harpreet Singh Grewal, Kuldeep Kumar Saxena, Kahtan A. Mohammed, Chander Prakash, J. Paulo Davim, Dharam Buddhi, Ramesh Raju, Dhanesh G. Mohan, Jacek Tomków

**Affiliations:** 1 Surface Science and Tribology Lab, Department of Mechanical Engineering, Shiv Nadar Institute of Eminence, Gautam Buddha Nagar 201314, Uttar Pradesh, India; 2 Department of Mechanical Engineering, GLA University, Mathura 281406, Uttar Pradesh, India; 3 Department of Medical Physics, Hilla University College, Babylon 51002, Iraq; 4 Division of Research and Development, Lovely Professional University, Phagwara 144001, Punjab, India; 5 Department of Mechanical Engineering, University of Aveiro, Campus Santiago, 3810-193 Aveiro, Portugal; 6 Division of Research & Innovation, Uttaranchal University, Dehradun 248007, Uttarakhand, India; 7 Department of Mechanical Engineering, Sree Vidyanikethan Engineering College (Autonomous), Tirupathi 517102, Andhra Pradesh, India; 8 Department of Material Processing Engineering, Zhengzhou Research Institute of Harbin Institute of Technology, Zhengzhou 450002, China; 9 Faculty of Mechanical Engineering and Ship Technology, Gdańsk University of Technology, 80-229 Gdańsk, Poland

**Keywords:** magnesium, aluminum, laser-based powder fusion, processing parameters, mechanical characteristics, post-processing approach

## Abstract

Complex structures can now be manufactured easily utilizing AM technologies to meet the pre-requisite objectives such as reduced part numbers, greater functionality, and lightweight, among others. Polymers, metals, and ceramics are the few materials that can be used in AM technology, but metallic materials (Magnesium and Aluminum) are attracting more attention from the research and industrial point of view. Understanding the role processing parameters of laser-based additive manufacturing is critical to maximize the usage of material in forming the product geometry. LPBF (Laser powder-based fusion) method is regarded as a potent and effective additive manufacturing technique for creating intricate 3D forms/parts with high levels of precision and reproducibility together with acceptable metallurgical characteristics. While dealing with LBPF, some degree of porosity is acceptable because it is unavoidable; hot ripping and cracking must be avoided, though. The necessary manufacturing of pre-alloyed powder and ductility remains to be the primary concern while dealing with a laser-based additive manufacturing approach. The presence of the Al-Si eutectic phase in AlSi10Mg and AlSi12 alloy attributing to excellent castability and low shrinkage, attaining the most attention in the laser-based approach. Related studies with these alloys along with precipitation hardening and heat treatment processing were discussed. The Pure Mg, Mg-Al alloy, Mg-RE alloy, and Mg-Zn alloy along with the mechanical characteristics, electrochemical durability, and biocompatibility of Mg-based material have been elaborated in the work-study. The review article also summarizes the processing parameters of the additive manufacturing powder-based approach relating to different Mg-based alloys. For future aspects, the optimization of processing parameters, composition of the alloy, and quality of powder material used will significantly improve the ductility of additively manufactured Mg alloy by the LPBF approach. Other than that, the recycling of Mg-alloy powder hasn’t been investigated yet. Meanwhile, the post-processing approach, including a homogeneous coating on the porous scaffolds, will mark the suitability in terms of future advancements in Mg and Al-based alloys.

## 1. Introduction

Provided that a product is constructed layer-by-layer from three-dimensional (3D) data, the additive manufacturing (AM) approaches are frequently referred to as layer-by-layer manufacturing [1,2,3]. Fabricating geometrically complicated objects by utilizing a variety of AM technologies are beneficial for industrial purpose [2]. These approaches can meet demands by lowering the time from design to manufacturing by substituting a single production procedure followed by a finishing step for several production procedures. Additionally, this satisfies manufacturers’ desire to reduce lead time and supply chain. Since only the material required to create the desired product is used, or waste is avoided, several AM approaches offer the benefit of potential raw material savings [3,4,5]. It encourages the creation of hybrid materials and cost-effective parts and products that can achieve functionalities that are currently not possible [6]. Businesses worldwide are prompted and benefitted from the advancement in the field of additive manufacturing. Despite its benefits, it cannot be assumed that it would be appropriate or practical for enterprises of all shapes and sizes without taking complexity, customization, and production volume into account. Depending on the particular AM approach under investigation, there are distinct tiers of advantages that designers can take advantage of through either the evolutionary design of already existing products or revolutionary approaches that impart functionality that is not possible with conventional manufacturing techniques [7]. The capacity to incorporate complexity that is largely cost-insensitive is one of the main benefits of using AM, which is why design flexibility is the main justification for its use. This includes the capability to provide design features that are not possible traditionally, such as embedding complicated internal structures or channels into designs (lattices); enabling light-weighting through topology optimization; and ultimately, the production of multi-material, multifunctional devices. Combining components into assemblies has another benefit, namely, reducing the need for mechanical fixtures and extra production processes [8].

Polymers, metals, and ceramics are just a few of the materials that can be used in AM technology. Among these materials, metallic materials are attracting more attention from the research and the industrial point of view. For instance, Debroy et al. [9] revealed metallic printed materials’ microstructures, flaws, and mechanical characteristics. Yakout et al. [10] depicted that the mechanical characteristics of various metallic alloys such as titanium and nickel alloy etc. were affected by the process parameters of the 3D printing approach. Mg alloys are promising degradable biomaterials used for orthopedics, cardiology, respiratory, and urology [9,10,11]. Since the device totally disintegrates, therefore long-term issues can be reduced or avoided with the usage of Mg-based materials. The key benefit of using Mg for orthopedics is that magnesium has an elastic modulus similar to bone, which reduces the negative effects originating from stress shielding [10]. Nevertheless, because of their relatively poor degrading characteristics (caused by the reactive nature of magnesium) and restricted formability, cast alloys have not been widely used in these applications (due to insufficient deformation modes and strong basal texture) [11,12]. Exploring the possibilities for alternate manufacturing methods to create the next generation of alloys with the desired physical qualities can therefore be important for a wide range of industries, from complicated lightweight consumer items to other developing technologies. For instance, the design flexibility of additive manufacturing can completely address the formability problem of Mg alloys since it allows for the manufacture of parts with near-net shapes and does not require further shaping or forming of the alloys [13]. Additionally, adjusting the process conditions can produce alloys with customized microstructures and property enhancements. The advancement of laser-based additively manufactured Mg alloys is discussed in detail marking the importance of lightweight complex parts and products formed to widen the usage of additive manufacturing.

Metal additive manufacturing technologies are evolving at a rapid pace due to the advancement of industrial applications that are easily accomplished with the usage and advantages offered by additive manufacturing approaches [14,15,16]. Due to their excellent fluidity, high corrosion resistance, and resistance to hot cracking, hypoeutectic aluminum-silicon alloys (such AlSi10Mg) are frequently utilized in light alloy materials [7,8]. Aluminum alloys are often manufactured using casting, forging, extrusion, and powder metallurgy. These processes typically have extensive production cycles and require a lot of work to fabricate composite materials, among other aspects. One additive manufacturing (AM) approach with a lot of potentials is selective laser melting (SLM) [14,15,16]. Layer-by-layer construction of a part allows for the rapid production of complex forms in an SLM process. SLM technology can successfully address the limitations of conventional manufacturing methods [15]. The SLM approach has a higher cooling rate that is precisely important in grain refining and obtaining better mechanical properties as compared with the conventional processing approach. The processing parameters of laser-based additive manufacturing are obtained to be critical to maximize the usage of material in forming the product geometry. With the advancement in technologies, additive manufacturing has become the pre-requisite technology for researchers and industrialists. With the present need to develop lightweight alloys with complex and customized product formation geometry, the work study revealed the advancements and recent developments related to laser-based additively manufactured Mg and Al alloys. The review article also highlights the number of alloys (Mg and Al) manufactured by the additive manufacturing approach. Currently, though, only a small number of Al and Mg alloys can be processed by laser-based additive manufacturing technologies. The related mechanical and tribological properties of Mg and Al alloy had been critically identified in the research studies. The challenges related to the fabrication of Mg and Al alloys were also discussed.

## 2. Additive Manufacturing Approach: Magnesium-Based Alloys

Magnesium alloys continue to be important in the context of modern and lightweight technologies. The increased use of Mg each year indicates a rise in demand for alloys containing Mg. With additive manufacturing (AM), components can be produced directly in a net shape, providing new ideas relating to the new prospects for Mg-based materials. The high feasibility of unique physical structures prepared by 3D printing widens the opportunities offering new advancements in additively manufactured Mg alloys. Magnesium (Mg) is the least dense of the engineering metals (1.74 g/cc), with densities that are roughly 65% lower than those of aluminum alloys, 38% lower than those of titanium, and 25% lower than those of steel [17,18,19]. Mg-based materials are desirable for lightweight applications in consumer electronics, aerospace, and automotive industries, resembling high specific strength [20]. With suitable biodegradability, the elastic modulus of Mg-based alloys is quite similar to that of natural bone i.e., 45 GPa [21,22] imparting protection against the stress shielding and providing sufficient healing to tissue. Mg-based materials are quite often found suitable for orthopedics in biomedical applications, such as fracture fixation, dynamic stability, joint replacement, cardiology, and maxillofacial applications [20,21,22,23]. Currently, casting (including precision die casting) accounts for more than 95% of the production of magnesium alloy products, whereas wrought magnesium alloys are only used in a restricted number of applications due to their poor formability and processability at room temperature [24,25].

**Figure 1:**
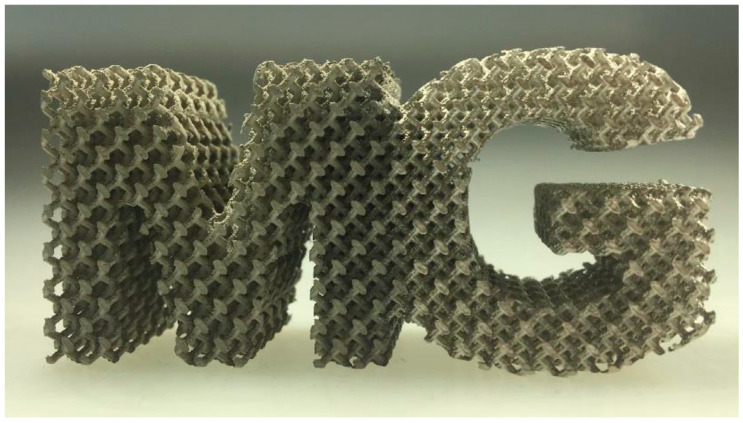
Laser powder-bed fusion (LPBF) created an Mg-shaped lattice structure in a magnesium alloy [27].

**Figure 2:**
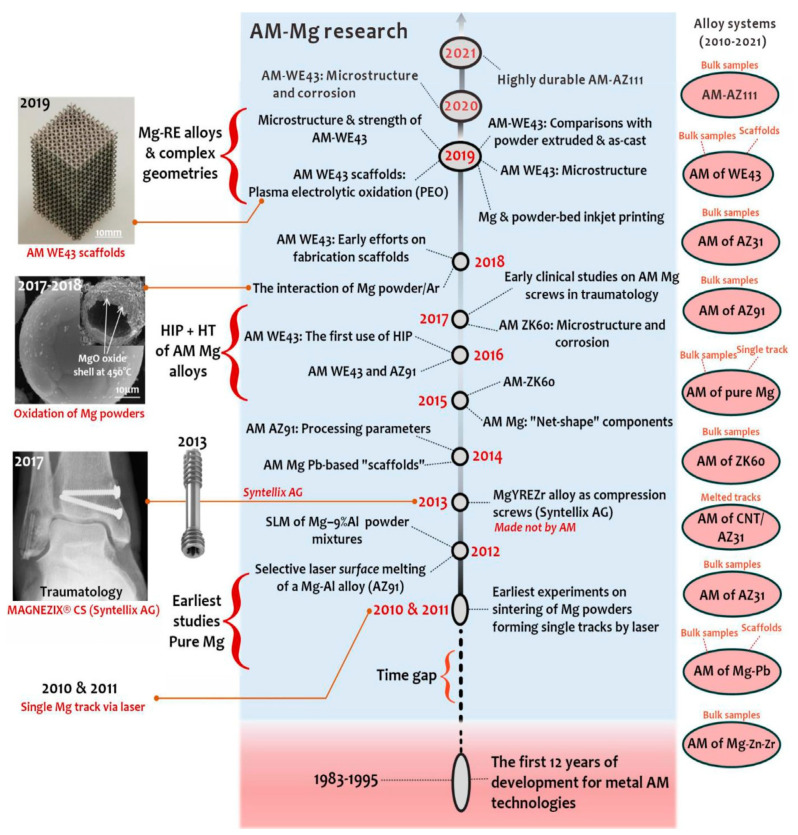
Advancement in the development of the Mg-based alloy via additive manufacturing powder-based approach [33].

Since additive manufacturing (AM) enables design capabilities that are not possible with traditional manufacturing and maybe also because material properties are still unknown, the interest in Mg alloys among the materials community is expanding. Additive manufacturing offers several exceptional benefits, including design freedom (topology optimization), little resource waste, and low energy consumption [26]. The drawback of traditional (formative or sub-tractive) fabrication routes is also eliminated by AM. The construction of precise geometrical characteristics such as those seen in Figure 1 is made possible by the capacity to produce complicated external and internal geometries with great accuracy [27]. With design flexibility, it is possible to optimize topology and use free space as a design variable to form the lightest engineering materials more-lighter. Furthermore, components having a big surface area, when utilized as biomaterials, enabled cell development, bone regeneration, and proliferation; alternatively, when employed as Mg electrodes, these components would offer a sizable reaction area [28]. The AM technique used for Mg-based materials is proved to be advantageous in fulfilling the rising demands for high-performance implants (biodegradable) for vascular and orthopedic surgery and making technological production more patient-specific and optimized the topological implants practically [27,29]. Additionally, the exact control of the process variables might result in alloys with custom microstructures and characteristics. Numerous AM techniques have been successfully used in recent studies to produce novel alloys with improved properties that are based on Al, Fe, and Ti [30,31,32]. However, there hasn’t been much research done on AM-Mg alloys thus far. This may be partly because magnesium is reactive under air circumstances, which presents questions about health and safety as well as handling, oxidation, and evaporation of Mg-based materials. The research study from 2010 entails the controlling of risk factors while persisting with Laser-Powder Bed Fusion (LPBF) as depicted in Figure 2. The LPBF approach of additive manufacturing is extremely effective in preparing additively manufactured Mg-based material products with greater accuracy by varying the compositions of the Mg alloys [27]. The required objective adheres to the current developments in AM-based Mg materials, thoroughly examining and evaluating the findings so far, and identifying the critical element that controls the overall characteristics of AM-based Mg materials. 

### 2.1. Laser-Based Additive Manufacturing Approach

The most extensively researched energy source for AM-Mg is a laser, which offers certain distinct benefits over other energy sources. To melt the powder, lasers (high concentration of heat) are concentrated over the specified area of the powder bed for a short time duration. The molten powder is rapidly heated and quenched by this short-duration heat flux, which promotes fast solidification. The most extensively used additive manufacturing technique for magnesium alloys is attributed to LPBF, often recognized as SLM (Selective laser melting). Only a very small number of studies related to AM-based Mg materials are attributed to DLD (Direct laser deposition). Today, the LPBF method is regarded as a potent and effective additive manufacturing technique for creating intricate 3D forms with high levels of precision and reproducibility together with acceptable metallurgical qualities [34,35,36]. Mg has an evaporation point of 1091 °C, while Al and Ti have evaporation points of 2470 °C and 3287 °C, respectively [37]. As a result, the temperature during LPBF will undoubtedly be higher than the temperature at which magnesium vaporizes, changing the composition of magnesium alloys generally. Systematically examining the evaporation during LPBF was done by We et al. [38]. It was discovered that the melting pool’s rising temperature greatly quickens the rate at which magnesium burns. Several processing parameters, such as laser power, scan speed, hatch spacing, and layer thickness, have an impact on the melt pool’s temperature. Porosity was regarded as the most critical issue while dealing with the LPBF approach for Mg-based material that needs to address, in addition to evaporation. Table 1 summarizes the impact of processing factors on the porosity of magnesium particularly. It was discovered by analyzing the reference papers that LPBF powders are categorized as mixed Mg and Al powder, rather than Mg-Al powders that have already been pre-alloyed [27,39,40,41,42,43]. This indicates that achieving high relative density utilizing mixed elemental powders (metal) may be more challenging, since the various thermal characteristics of each element may generate substantial local incompatibility in rapid cooling.

While some degree of porosity is acceptable because it is unavoidable, hot ripping and cracking must be avoided. The most serious problems that lower the as-built component’s quality in LPBF are hot tearing and cracks [63,64,65]. In general, low constitutional supercooling gives rise to the formation of columnar grains, but the temperature gradient is still substantial, making them particularly susceptible to hot ripping. Along with volumetric shrinkage during solidification, the thermal contraction between the columnar grains, attributed to hot tearing and cavities formation, results in enhancement in the length of columnar grains when temperature and liquid volume fraction drop [66]. No evidence related to the effects of processing parameters and alloying elements on hot tearing evolved in Mg-based materials advancing to the LPBF technique. Therefore, as per the future aspect, the significance of alloying elements might be considered a better option along with processing parameters identifying the behavior of tearing in Mg materials. Empirically, an alloy (Mg-6Zn) that has columnar grains and entails a high solidification range might be considered more vulnerable to cracking [67,68,69,70]. Furthermore, research can be accomplished in evaluating the fracture mechanism of additively manufactured Mg-based material identified as a function of process parameters of LPBF and the composition of the alloy.

### 2.2. Investigation of Mg Alloy via Additive Manufacturing

Advancing to additive manufacturing approach for Mg-based materials, very few combinations have been studied when compared to wrought and cast alloys. This is primarily due to the high expense of producing atomized pre-alloyed powder on a customized basis, which is highly expensive compared with the customized composition of wrought and cast alloy. Pure magnesium, AZ91, and WE43 are now the most widely used compositions of magnesium-based materials used for additive manufacturing [71,72,73,74,75,76]. These alloys are attributed to superior printability, sustainability in structural and biological applications, and attracting market demand (for being lightweight). The detailed research outcomes of the various research studies have been compiled in the section below to identify the development relating to the AM approach to Mg alloy. The study paved a way for future research related to additively manufactured Mg-based materials.

#### 2.2.1. Pure Mg Alloy

**Figure 3:**
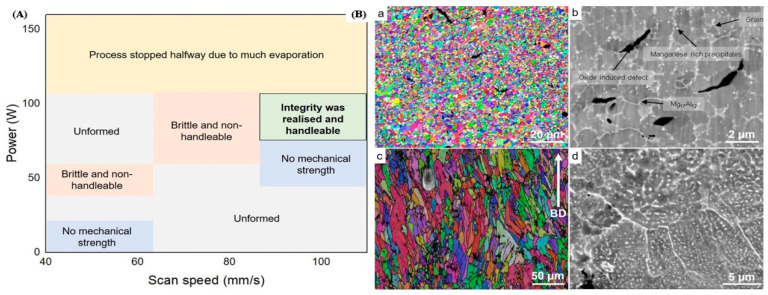
(**A**) Processing parameters of laser-based powder approach, (**B**) (**a**–**c**) depicted EBSD orientation, (**b**–**d**) SEM characterization of AZ91 alloy formed by laser powder bed fusion [27].

At the initial stage the researcher, Ng et al. examined the first experimental approach to produce customized equipment using a laser-based additive manufacturing approach relating to Mg-based material [77]. The Nd-YAG laser was used primarily as a source of heat to melt the powder over the powder bed in the LPBF approach. For a single-track laser scan, several laser powers and scan speeds were tested during the initial research relating the Mg-based material to the LPBF approach. The variation of laser power with scan speed was depicted in Figure 3A [77,78]. It was concluded from various research studies that the LPBF approach of additive manufacturing does not succeed with irregular and coarse powder. Other than that, the LPBF approach holds good accountability with spherical and atomized fine powder under the pre-requisite condition of processing parameters [77]. The variability of grain size with pure Mg obtained via LPBF was observed in the range of 2 to 5 μm. To incorporate such a tiny grain size in pure Mg is quite a difficult task before advancing to the LPBF. Only an extreme plastic deformation approach at low-temperature conditions was able to accommodate the tiny grain size distribution in pure Mg materials [79,80]. Therefore, the LPBF approach plays a significant role in refining the microstructure of material over traditional casting and thermomechanical processing. Furthermore, research studies identified that the LPBF single-track sample has an extremely high hardness as well as a significant density of cracks around the grain boundaries and formed the oxide layers around the boundary [81,82,83]. The researcher Hu et al. developed the first bulk Mg relating to the LPBF approach used in producing customized parts of Mg material. For the spherical shape of powder, the high density of gas pores was obtained through the LPBF approach, while the irregular shape of powder marks the abundance of fusion pores but resembles the structure depicting certain interconnectivity between the pores [84,85]. Pure Mg material relating to the additive manufacturing approach can be produced by the DLD approach (Direct laser deposition) of additive manufacturing in addition to the LPBF approach [86,87].

#### 2.2.2. Mg-Al Alloy

The most significant commercial composition of Mg-Al-based alloys in the cast and wrought forms is AZ31 [88,89,90]. While the majority of the formation of AZ31 alloy-based additively manufactured parts is attributed to the wire-arc approach but there is very little literature on laser-powder-based additive manufacturing. In actuality, high-Al concentration Mg-Al alloys, such as AZ91, make up the majority of laser-based Mg-Al alloys [91]. This is because the addition of Al necessitates grain refinement of the alloys through super-heating or inoculation, enhances castability (hence printability), and offers reinforcement through the solute and β-Mg_17_Al_12_ intermetallic phase [91,92]. Coming to the LPBF approach, Pawlak et al. investigated the fabrication of AZ31 alloy-based material parts via the LPBF approach and attributed it to the low porosity level of around 0.5% [93]. In LPBF, AZ61 and AZ91 also attain such a low porosity level, proving the alloy’s acceptable printability [27,94]. The AZ91 and AZ61 alloys forming through the LPBF approach attributed to equiaxed and fine grain distribution, as well as attaining the texture that was almost randomly distributed [95,96,97,98]. Figure 3(Ba) marks the variation in the grain size distribution ranging from 1 to 3μm in Mg-Al alloy prepared via the LPBF approach. According to some research, the β-Mg_17_Al_12_ intermetallic is primarily absent from the grain interior and is instead scattered along the grain boundaries and linked, as seen in Figure 3(Bb) [27]. However, some results display grains that are extended in the construction direction seen in Figure 3(Bc). While the intermetallic phase (β-Mg_17_Al_12_) finds around the grain boundary. Furthermore, the research identified that there exist abundant spherical intermetallic (β-Mg_17_Al_12_) nanoparticles inside grain boundaries attaining a diameter of around 300 nm as identified in Figure 3(Bd) [27].

#### 2.2.3. Mg-RE Alloy

**Figure 4:**
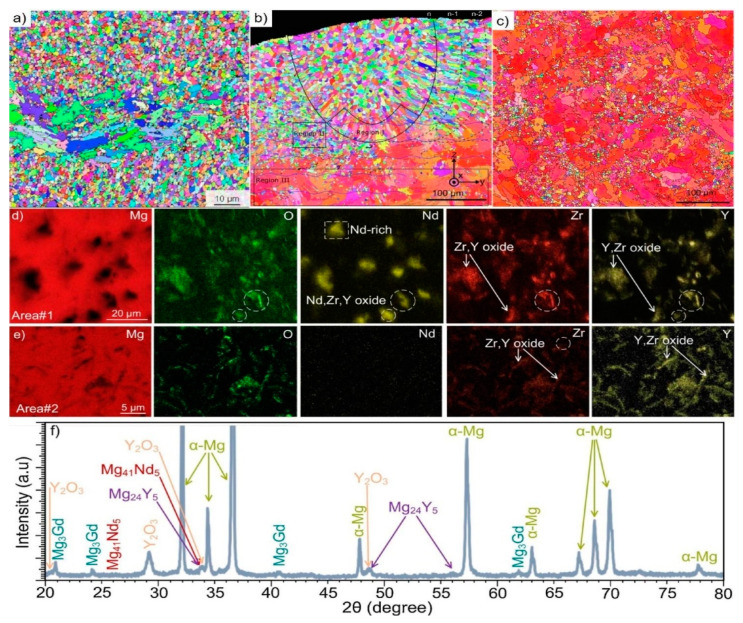
EBSD image shows equiaxed, fine, and random grain representation in (**a**) bulk LPBF-WE43, (**b**) last melt pool corresponds to basal- orientated, large, and irregular shape grains, (**c**) basal- orientated, large, irregular shape grains in the bulk sample, (**d**,**e**) EDS image at different magnification for same materials, and (**f**) XRD image depicted intermetallic and oxygen-rich elements in WE43 alloy [27,33,56].

The additively manufactured Mg-RE alloy has received the greatest attention for use in biomedical implants especially WE43 alloy. Although WE43 alloy attains significant importance in biomedical applications, it also has good printability which accounts for a new doorway in order to achieve the reduced porosity [99,100], better than AZ91 as discussed in [93]. Being biocompatible, WE43 alloy does not cause any negative cell reactions such as cytotoxicity, whereas Aluminum in Mg-Al alloy accounted for cytotoxicity. Al is a neurotoxic element that is prohibited from bioabsorbable magnesium alloys due to the high concern relating to Alzheimer’s disease. Consequently, WE43 alloy has garnered increased interest as a biodegradable implant material in scaffold applications [27]. Despite, very few grains with aberrant grain development during LPBF, Zumdick and Jauer’s early tests of LPBF over WE43 showed the formation of equiaxed grains around the boundary and provides a pathway to the refinement in the grain size, depicted in Figure 4a [27]. Intriguingly, the LPBF approach over WE43 alloy exhibited a completely different microstructure way back in 2019 pertaining to the similar processing parameters illustrated by the same research team, entailing the dominance of large, strongly basal-textured grains with irregular shapes [56]. Figure 4b–d shows that although the laser beam’s quick solidification of the melt pool produces fine, columnar, equiaxed grains [33,56]. The succeeding laser scans in the LBPF process result in heat treatment, which leads to grain development with a distinct [0001]/BD texture. It is demonstrated that, following a single-layer deposition, there is significant grain development and that, following the formation of two layers, the grains achieve their maximum size. It is uncertain what precise mechanism results in such vast grain expansion and textural development. In contrast, no such grain development can be seen in Figure 4a [27]. The authors suggested that yttrium oxide (Y_2_O_3_) particles, which are thought to offer Zener-pinning to inhibit grain formation, may be present in varying proportions in powders from various vendors [101,102]. In actuality, oxygen and the early RE elements have a strong affinity towards inhibiting the formation of grains. In comparison to MgO (596 kJ/mol), the Gibbs free energy required for the production of Y_2_O_3_ and Nd_2_O_3_ is 1815 and 1806 kJ/mol, respectively [27,103]. Therefore, a significant proportion of RE oxide has been present in all LPBF-WE43 publications to the date shown in Figure 4e,f [56]. The big and basal-oriented grains are nonetheless predominant in the WE43 alloy formed by the LPBF approach concluded by Esmaily et al., despite the high density of RE oxide that doubts the efficiency of RE oxide in preventing grain development from Zener Pinning [104,105]. In actuality, the concentration and types of solute particles often referred to as the Growth Restriction Factor proposed by St John, have a greater impact on grain growth during solidification [106,107]. The Growth Restriction Factor (Q) in this model is

Q = C_0_m(k − 1)

where K relates to the equilibrium distribution coefficient, C_0_ related to the composition of solute particles, and m relates to the liquidus line slope.

A larger solute concentration causes a greater thermodynamic limit on grain development that has been observed in various research studies [108,109,110]. Therefore, the low concentration of solute particles relating to the RE element in the powder prevents the oxidation of WE43 alloy powder during production, transportation, and storage. Due to the inability of the low solute particle concentration in powder to prevent preferred development, the result is the massive, basally oriented grains as seen in Figure 4b,c [56]. Therefore, researchers have customized the composition of powder and inherited various compositions resembling the Mg-Gd systems in addition to experiments based on commercial WE43 powder [111,112]. The as-LPBF Mg-Gd-based alloy incorporated the significant grain refinement (1–2 μm), resembling equiaxed grain with the random distribution of grains around the boundary [27]. The relative density of the alloy can reach 99.95%, and it has few oxides and pores. Similar behavior was obtained in DLD (Direct Laser Deposition) manufactured Mg-10Gd-3Y-0.4Zr alloy with spherical powder (100–300 μm) pertaining to the randomness in the distribution of the equiaxed grains [27]. DLD reveals that the alloy sample has a bigger grain size and a higher pore percentage. Therefore, in order to limit the enhancement in the grain size of basal-oriented grains, the appropriate amount of Gd content (>10 wt.%) should be primarily used during solidification, irrespective of any approach used (DLD or LPBF) [113,114].

#### 2.2.4. Mg-Zn Alloy

Despite Zn being biocompatible in nature, Advancement to Mg-Zn alloy has not been explored significantly as comparable to Mg-RE and Mg-Al alloys. The research studies suggested that the wide range of solidification and low eutectic temperature (325 °C) of Mg-Zn alloy, accounts for the poor printability as compared with Mg-RE and Mg-Al alloys [27]. Only at very low (less than 1 wt.%) and very high (12 wt.%) concentrations of Zn will produce an acceptable level of porosity. Resembling ZK60 alloy where Zn concentration opt at 6 wt.%, the hot cracking and higher density of pores were accommodated in the additively manufactured ZK60 alloy [115,116]. As a result, the alloy is rendered useless and unusable. The research studies concluded that ZK60 alloy produced by the LPBF approach produces a relative density of around 97%. Therefore, the addition of Zn as an alloying element in the additively manufactured Mg-based materials via a laser-based approach adheres to the minimal quantity. In addition to the Mg-Zn, Mg-RE, and Mg-Al-based alloys, the research studies explored the Mg-Sn alloy with the blended powder and Mg-Ca alloy with pre-alloyed powder [27]. The outcomes depicted the short range of solidification and high value of eutectic temperature (466 °C and 510 °C for Mg-Sn and Mg-Ca alloy system) which accounts for the higher printability of these alloys as compared with Mg-Zn alloy. Along with good printability, these alloys incorporated the equiaxed grain and prompted the refinement of the microstructures [117]. But for a future perspective, more research needs to be carried out on these alloys via LPBF identifying the behavior of solidification, the evolution of the microstructure, and the mechanical and electrochemical properties [118,119,120,121,122].

### 2.3. Mechanical Properties of Laser-Based Additive Manufacturing Approach

**Figure 5:**
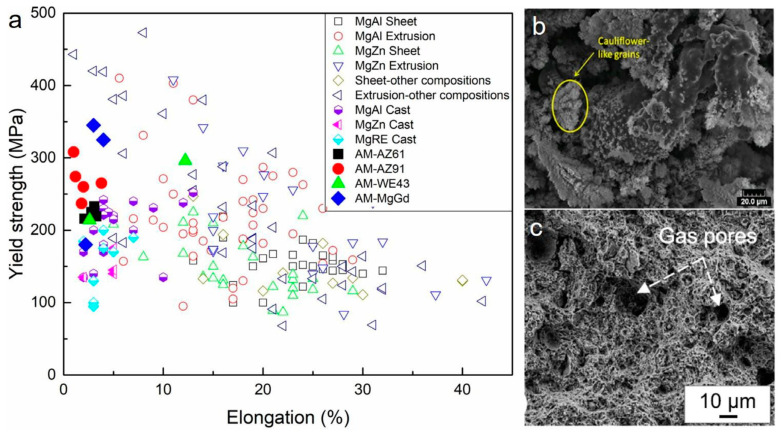
(**a**) Tensile characterization of additively manufactured Mg-based material via LPBF approach against wrought and cast alloys, (**b**,**c**) Fractured surface of (**b**) Mg-9Al alloy and (**c**) WE43 alloy [27,54,46].

Accounting for the mechanical characteristics of additively manufactured Mg materials with a laser-based approach, the research outcomes are concluded in Table 2 for future research perspective. The graphical variation in the yield strength with elongation (%) for various wrought alloys (extruded and rolled) and cast alloys is shown in Figure 5a [27]. For laser-based additively manufactured parts, the compression or hardness test are pre-requisite in order to analyze the mechanical behavior of the AM-Mg alloy as prepared parts via LPBF account for the ductility of less than 5%, while some of the alloys have none at all which is unacceptable for engineering material. Other than low ductility, some alloys pertain the weak texture, or equiaxed, fine grains, and resemble low porosity during the microstructure behavior, irrespective of low ductility [123,124,125]. Low porosity accounts for good printability of the alloying material. Furthermore, research studies were focused in order to identify the reason for low ductility in laser-based additively manufactured Mg materials. Firstly, the quick solidification causes the as-LPBF to have significant residual stress, which lowers the alloy’s ductility [124]. Secondly, the examined alloys such as Mg-Gd, WE43, and AZ91 alloys, include significant amounts of alloying elements addition incorporated in the intermetallic phase around the grain boundaries. Therefore, due to the formation of the intermetallic phase around the grain boundary, the brittle behavior as well as local failure around grain boundaries were observed. The presence of local failure showcases the inability of material to cause the plastic deformation (twining and slipping around the boundary as well as sliding of grain boundary etc.). Low ductility encountered in the laser-based additively manufactured part was due to the poor redeposition of powder or vapor over the surface of parts that weakened the bond between the particles. The fracture surface’s cauliflower-like characteristic is shown in Figure 5b. The WE43 alloy currently has the highest documented ductility among laser-additive-produced magnesium alloys at 12.2% [27]. Despite the presence of some gas pores, the fracture surfaces were clearly visualized in Figure 5c that the sample has broken in a ductile manner. A high-temperature annealing approach can increase the ductility of an alloy. The enhancement in the ductility of WE43 alloy formed by LPBF encountered 2.5% in the as-built state to 4.5% after heat treatment by annealing at 535 °C for 24 h and aging at 205 °C for 48 h [126]. FSP (Friction stir processing) dramatically reduces the residual stresses, and grain size, and redistributed the intermetallic of Mg-10Gd-0.3Zr alloy, leading to a more striking increase in ductility from 2.2 to 7.5% [27]. Although it is unlikely that the net shape component formed via the LPBF approach will be produced by the friction stir processing approach in actual applications of engineering showcasing that the alloy formed by the LPBF approach is not inherently brittle in nature [127]. Therefore, the optimization of processing parameters, composition of the alloy, and quality of powder material used improves the ductility of additively manufactured Mg alloy by the LPBF approach. The detailed description of the investigation of mechanical characteristics of Mg-based alloy prepared by powder-based fusion approach of additive manufacturing.

### 2.4. Electrochemical Durability of Mg-Based Alloy Prepared by Lased-Based Powder Fusion

Biodegradable implants are attributed to the most promising aspect of additively manufactured Mg-based materials. For better implantation outcomes, oral and maxillofacial implants retained sufficient mechanical integrity for the initial first month before gradually deteriorating, becoming completely dissolved and metabolized after three months [128]. Given that magnesium and its alloys are known to have low corrosion resistance in the majority of aqueous settings, this demands adequate electrochemical durability. With regard to the LPBF approach, the corrosion current density (I_corr_) of Mg (pure) in Hank’s solution is far better than the cast Mg (pure) ingot tested under the same conditions (23.6 µm/cm^2^) and varies from 74 to 177 µm/cm^2^ [129]. Depending on the processing conditions, the mass loss rate ranges from 3 to 32 mm/year. In a solution of 3 wt.% NaCl, the corrosion rate of pure Mg produced by DLD is about 144 mm/year [27]. The loosely fused Mg clusters and sintered Mg powder provide a negative effect attributing to corrosion resistance. As a result of the higher corrosion rate, the parts formed by the LPBF approach inherited some defects, advancing localized corrosion [130]. The rate of degradation increases with the number of faults and defects in the parts formed. Similar to the cast alloy, the LPBF WE43 alloy displayed significantly less corrosion resistance. In r-SBF solution (revised simulated body fluid) containing fetal bovine serum up to 5%, the corrosion current density varies from 20 to 60 µm/cm^2^, and in a solution of 0.1 M sodium chloride, the mass loss rate is approximately 6 times greater as compared with cast WE43 alloy (0.8–1.2 mm/year) [56,131]. Irrespective of higher relative density (<99%), the micro galvanic reaction, attributed to a high density of RE oxide and reactive magnesium matrix, resulted in an improvement in the rate of corrosion [132]. If the surface of the LPBF-WE43 scaffold is not exposed to PEO (Plasma electrolytic oxidation), it has been reported that the structural integrity of the scaffold will lose after 21 days of immersion in simulated body fluid (SBF). The research studies concluded that the corrosion resistance of the cast alloy is superior to that of the Mg-Al-based alloy. The degradation rate for AZ61 alloy formed by the LPBF approach was approximately 6 to 8 mm/year during the state of as-immersion, and subsequently, it decreased and gets stabilized in SBF, reducing the degradation rate to about 1.2 to 2.7 mm/year [27]. The aforementioned degradation is comparable to the cast AZ61 alloy in SBF depicting the rapid rise in the rate of corrosion to around 6.5 mm/year, but slowing down to 1.299 mm/year after 24 days of immersion [133,134]. The research data concluded that ZK60 alloy formed by the LPBF approach provides superior corrosion resistance as compared with cast ZK60 alloy, based on the hydrogen evolution rate and corrosion current density data [135]. Apparently, the surface of the ZK60 alloy formed by the LPBF approach indicates a more severe corrosion rate [27]. By combining ZK powders with Cu powders, Shuai et al. increased the antibacterial activity of Mg-Zn-Zr implants by adding diluted concentrations of Cu to ZK30 and ZK60 alloy formed by the LPBF approach [27]. It was concluded that the LPBF ZK-Cu alloy formed by the LPBF approach degrades more quickly when Cu is added. Therefore, Cu serves as the suitable alloying element to control the degradation rate of the Mg-Zn-based alloy system.

### 2.5. Biocompatibility of Mg-Based Alloy Prepared by Lased-Based Powder Fusion

The biocompatibility of LPBF-Mg alloys must be taken into account because biodegradable implants are the most promising application for AM-Mg alloys. Being the crucial component of the human body, the degradation rate of magnesium-based material shifts the stresses from the implant to the rebuilt bone. Mg-based materials are equivalent to human bone in terms of both density (1.7 g/cm^3^) and young’s modulus (45GPa) [136]. Mg is both biocompatible and bioactive, which considerably encourages cellular division and proliferation [137]. The stabilization of RNA and DNA, as well as bone formation and healing, all benefit from it. Therefore, the biocompatibility of the alloying components added to the Mg-based materials attributing to the biodegradable implantation. Furthermore, the research studies depicted that the neurotoxicity of aluminum ion (Al^3+^), attributing to the accumulation of these ions in the nervous system, resulted in Alzheimer’s disease. Al addition can increase printability such as Cu, which may have some antibacterial effects but is primarily cytotoxic [138]. Therefore, it is doubtful that alloys comprising Al and Cu will be found suitable for clinical application. Numerous research has so far confirmed the in-vitro biocompatibility of WE43 alloy formed by LPBF as a scaffold implant [56,70,139]. Although RE-based magnesium alloys themselves don’t appear to have any cytotoxic potential. The extensive reactivity of the bare metal surface is attributed to the high evolution of hydrogen gas. The high evolution of hydrogen gas leads to the shifting of pH, which interferes with cell metabolism [27]. Only a few dead cells could be seen after direct live/dead staining, and no viable cells could be seen on the WE43 alloy formed by LPBF for scaffold applications [139]. The conclusive evidence for surface modification, such as plasma electrolytic oxidation, can address this problem since it slows the production of degradation by-products and, as a result, encourages hardly any evidence of cell damage [140]. Passivating ceramic-like surfaces also appear to provide a good option for adherent cells [141]. In addition to WE43 alloy, it was reported that the LPBF scaffold was also made using a pre-alloyed system of Mg-Nd-Zn-Zr, commonly referred to as JDBM [27]. Comparable to WE43 alloy formed by the LPBF approach, the research study obtained by cell adhesion test identified that dicalcium phosphate dihydrate coating over the scaffold attributed to the generation of more cells that attached to the scaffold rather than uncoated scaffold [27]. Neither the coated JDBM scaffold nor the uncoated JDBM scaffold formed by LPBF contributed to any significant difference in the assessment of cytotoxicity. As a result, both samples promoted cell proliferation. From the research perspective, it was quite unacceptable that the uncoated sample of additively manufactured Mg material will not at least irritate direct cell response, hence this finding requires a more thorough investigation and verification in the future.

### 2.6. Challenges Inherited in Laser-Based Approach Relating to Mg-Based Material

The necessary manufacturing of pre-alloyed powder is difficult with regard to the laser-based additive manufacturing approach. Evidence, however, points to the suitability of combining elements with a combination of pre-alloyed powders. Further research is needed in the area of consistency and blending of magnesium powder. To fully comprehend the physical characteristics of Mg alloys prepared by AM, mechanistic studies are still needed. Undoubtedly, laser-prepared AM alloys show distinct characteristics on comparing to non-AM Mg alloys but the physical basis resembling such differences is still open (i.e., the impact of additive manufacturing on ductility and strengthening mechanisms). While addressing to laser-based additively manufactured approach relating to Mg-based material, ductility remains to be the primary concern [56]. It is recommended to have the smallest amount of powder while dealing with LPBF. However, the handling and storage of powder should be kept away from the ignition, limiting atmospheric exposure. Research findings also revealed that there exists a research gap in relation to the recycling of Mg-alloy powder that hasn’t been investigated yet. Furthermore, compositional and process parameter modification has not yet been researched. The sintering-based approach is a new technique that needs to be explored relating to Mg-based materials. More work can be accomplished on Mg-based material by binder jetting approach. There is a need to look into the post-processing approach, including a homogeneous/uniform coating on the porous scaffolds relating to Mg-alloys that has not been investigated.

## 3. Additive Manufacturing Approach: Aluminum and Its Alloys

**Figure 6:**
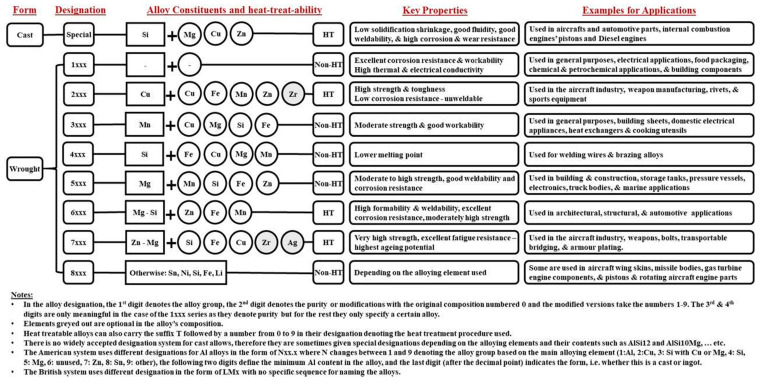
Major classification of Al alloy, highlighting the key properties and application [145].

Aluminum alloys are highly used in industrial applications due to their high performance, light-weight, and low costs with a good balance between strength and density. The family of aluminum alloys is categorized into various groups depending upon the heat-treated ability and primary alloy elements and shape of the alloying elements which are listed in Figure 6. In the current scenario, additive manufacturing relating to aluminum material incorporates all the industrial applications from the aerospace to automotive sector. The additive manufacturing approach, SLM (Selective laser melting) can be used to produce open-cell and bulk structures [142]. An aluminum alloy that is cellular or porous is a deformable, lightweight metal serving the purpose of crumple zone in automobile applications [143]. Aluminum-based materials that are difficult to process can be easily formed by SLM, retaining the shape benefits [142,144]. They resemble the AA-6xxx series, which is difficult to produce due to its abundance of hard intermetallic materials and can be formed by the SLM approach [145,146]. The microstructure of Al-based material i.e., cast alloy, gets refined and improved persisting to the SLM approach illustrating the advantage of the SLM approach [147]. In previous research, it was obtained that the modification of microstructure attributed to the improvement in the strength of the cast Al alloys [148,149,150,151]. SLM approach offers the refinement in microstructure relating to high cooling speed in SLM without altering the chemical composition during manufacturing [145]. As a result, the requirement for the manufacture of complicated structures with precise microstructures can be satisfied by the SLM processing of castable aluminum alloys. The majorly used SLM approach is described in the below section, along with the microstructure and mechanical characteristics related to the SLM approach of additive manufacturing used to prepare Al-based alloys.

### 3.1. Selective Laser Melting Approach Relating to Al Alloy

Al-based materials are difficult to process, but the SLM approach provides the desired way to process Al alloy, advancing to low absorptivity of laser relating to continuous or modulated fiber lasers, attributing to high thermal conductivity and reflectivity [152]. Cast alloys often serve as the most promising aspects of the SLM processing technique, indicating that AlSi10Mg and AlSi12 alloys are among the promising alloys [153,154]. The presence of Al-Si eutectic phase in AlSi10Mg and AlSi12 alloy attributing to excellent castability and low shrinkage, attaining most of the attention in the SLM approach. The Al-Si alloy offers high tensile characteristics and low ductility (4%) that are regarded as advantageous to Al-based material. The most frequently used high-strength Al alloys used in automotive and aerospace industries are 2XXX, 6XXX, 5XXX, and 7XXX, offering increased ductility [145,146,147,148,154]. However, regardless of improved ductility and high strength, the fabrication of these Al alloys is often difficult via the SLM approach. The formation of micro-cracks depicted on the surface of the Al-based material formed by the SLM approach due to rapid cooling persisting in processing and forming the piece attaining the low structural integrity [145,155,156]. From a research perspective, the evaluation of mechanical characteristics and microstructure of various Al alloys has been formed by a laser-based additive manufacturing approach in order to illustrate the research outlook for the future in the SLM approach.

### 3.2. Properties Evaluation of Al Alloy Formed by SLM Technique of Additive Manufacturing

Since it is a pre-requisite to analyze the mechanical characteristics of the Al-based materials in order to evaluate the viability of the SLM approach that is attaining the research popularity. The microstructure refinement in the SLM approach is governed by rapid solidification and material-laser interaction, attributing to an improvement in the material qualities. However, simultaneously, the presence of defects adhering in the Al-based materials during the processing condition of the SLM approach attributed to a negative effect on the mechanical behavior of the Al alloy formed by SLM. Therefore, by optimizing the process parameters of the SLM approach, the mechanical behavior of Al alloy can be improved, evolving the reduction in the defects in the micro-structure of the Al-based materials. The variation in the processing parameters of the SLM approach influences the anisotropy of material, attributing to the different crystallographic textures of Al-based materials [145,157]. The research studies concluded that build direction had a positive effect on the density of dislocations, attributing to better mechanical characteristics for AL-based SLM materials [158]. For a detailed evaluation of the mechanical characteristics of laser-based Al alloy, the research studies were highlighted in the below section. 

#### 3.2.1. Nano-Hardness of Laser-Based Additively Manufactured Al Alloy

**Figure 7:**
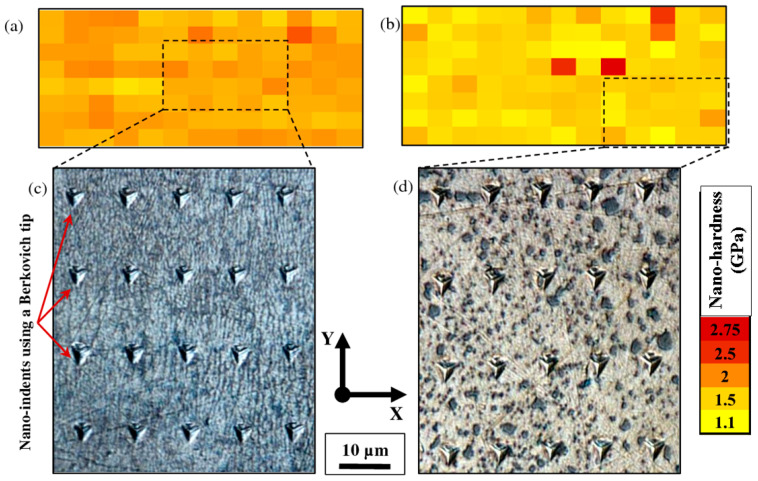
Nano-hardness image of an AlSi10Mg alloy fabricated on cast AlSi12 substrate depicted the homogeneity in the SLM material vs the non-uniform profile in the related part; Comparison in the nano-hardness of (**a**,**c**) the as-built material, and (**b**,**d**) the heat-treated material [161].

The highly precise hierarchal microstructures obtained by the selective-laser melting approach sparked the interest in researching the material’s local mechanical characteristics at the nanoscale level [145]. The research outcomes revealed that a uniform profile of the melt pool in AlSi10Mg alloy at the nano-scale is obtained with a depth-sensing indentation approach, pertaining to the improvement in the hardness of the alloy as compared with cast materials [159,160,161]. Everitt et al. entail that the similarity in the hardness of cast substrate and SLM-based materials supports the improvement in the hardness at the nanoscale attributed [145], resembling the uniformity in the melt pool. Similar findings had been recognized with SiC as a reinforcement in AlSi10Mg alloy by Zhao et al. attributing to the improvement in the uniformity in nano-hardness of alloy between molten pool core and boundary [162,163]. The extremely fine microstructure and the fine dispersion of the alloying components were both credited with the uniform profile in the material formed by the SLM approach [164]. In contrast, the cast material’s coarser microstructure displayed spatial variation that was dependent on the indentation phase [165,166]. The researcher Everitt et al. analyzed the consistency in nano-hardness inculcating the overlapping of melt pool in the single layer, attributing to the uniformity in nano-hardness across the multi-layer sample that was showcased in Figure 7a,c [161]. The research studies concluded that the researchers could infer that the local mechanical properties of the material are not significantly impacted by the overlap of the melt pools used to create the 3D structures. As the solidification and re-melting of the material do not enhance the nano-hardness of the material irrespective of grain size variation in each melting pool [167,168]. As depicted by Qi et al., the homogeneity of the material indicated by the nano-hardness profile, depending on the melting mode, obtained the variation in the mechanical characteristics of AA-7050 [145]. Therefore, a nano-hardness profile is regarded as the most crucial parameter in establishing uniformity in microstructure, attributing to the improvement in the overall mechanical characteristics of the materials. As per the research studies, melting in the conduction mode is attributed to the improvement in nano-hardness (higher nano-hardness), resulting in more uniformity in the hardness profile. The improvement in the area around the grain boundary with the presence of fine grains relating to the increase in nano-hardness at the bottom of the melt pool improves the overall mechanical characteristics of the material [169]. Figure 7b,d illustrates how the local mechanical properties of the material are impacted by the microstructural changes caused by heat treatment [161]. The spheroidization of silicon and thermal treatment of the silicon particles that resulted in their coarsening caused a spatial variation in the nano-hardness of the material, with improvement in hardness being attained by the coincident indentation on silicon particles [170]. Therefore, Nano-hardness obtained by the SLM approach in Al-based material is considered an important property in order to improve the mechanical characteristics and microstructure of the material used.

#### 3.2.2. Micro-Hardness of Laser-Based Additively Manufactured Al Alloy

**Figure 8:**
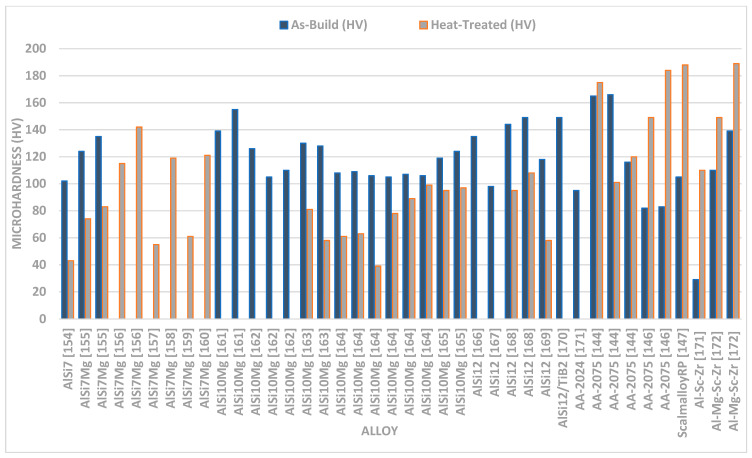
Variation in micro-hardness of Al-based materials formed by SLM approach under as-built and heat-treated condition.

**Figure 9:**
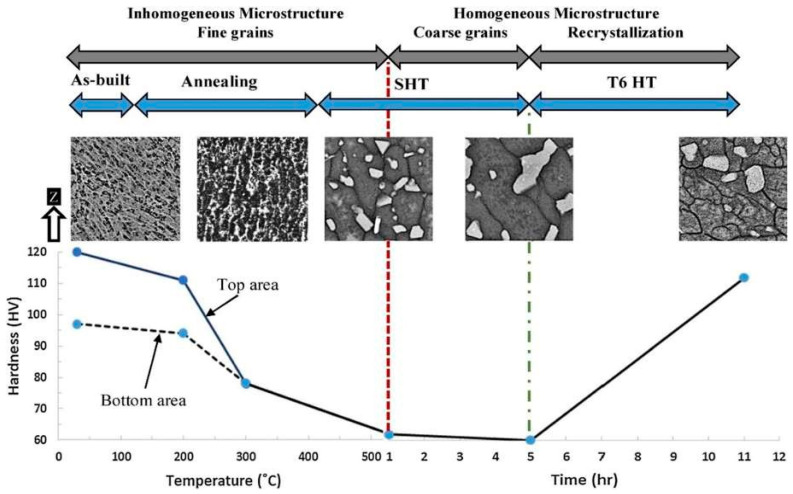
Variation in the microstructure of AlSi10Mg alloy relating the micro-hardness depending on the heat treatment process [171].

Due to the relative simplicity of the test and a large number of small samples, micro-hardness is frequently used to examine the mechanical characteristics of SLM Al parts [171]. This makes it a rapid technique to evaluate mechanical characteristics, in order to widen the application field of Al alloy formed by the SLM approach. Enhanced micro-hardness for SLM AA-2XXX has been reported in comparison to its traditionally produced counterpart alloys. The as-built AA-2024 alloy has a higher value of micro-hardness (greater than 37%), which is far more than the 2024-O sheet but, at the same time, 20% lower as compared with the T6-treated traditionally-processed sheet [145]. The major research finding up to date relating the microhardness of the Al alloy formed by the SLM approach is depicted in Figure 8. The variation in microhardness resembles certain factors of the SLM approach i.e., the processing parameter of SLM, the composition of an alloying element, and powder quality attributing to the variation in the densification factor. The mapping in Figure 9 demonstrates how the material was greatly softened by the different heat treatment techniques and involved solution heat treatment followed by aging, solution heat treatment, and annealing [171]. The research studies depicted similar trends of microhardness and nanoindentation segment. The authors took into account the different alloying elements that strengthened the AlSi10Mg samples in the condition of as-built and after heat treatment [145]. The strengthening of AlSi10Mg alloy is caused by the solid solution strengthening, dislocation strengthening, and grain refinement attributing to the obstruction in the dislocation motion inherited in the material dislocations obstructing one [172]. The main difference between the as-built and heat-treated materials is that the heat treatment coarsens the grain size attributing to the formation of Si spheroids which is revealed by the Orowan strengthening criteria [173]. Inculcating the influence of grain refinement in the microstructure, the microhardness of the as-built materials is far better than the heat-treated material. Therefore, softening of the material occurring in the annealing process due to the reduction in the dislocation density is attributed to dislocation annihilation and coarsening of the microstructure in the heat treatment [145]. The research studies identified that AA-7075 contributed to the remarkable result of the SLM approach, improving the properties of the materials [145,156]. For Scalm alloy RP, the creation of a significant amount of Al3Sc prevented material softening during the T6 heat treatment, resulting in an improvement of 69% of micro-hardness [147]. Additionally, it was claimed that Zr additions to Al alloys, either with or without Sc resulted in similar behavior as shown by Scalm alloy RP [174,175,176]. Takata et al., improvised the size of the sample from 0.1 mm to 10 mm, in order to analyze the variation in the microhardness behavior of AlSi10Mg alloy formed by the SLM approach [177]. Based on the research studies, it was analyzed that the particle dispersion in the matrix material entails the improvement in the local hardness of about 2–3 times greater as compared with the non-dispersion of particles, subtending with the passing of indentor with the brittle phases. Zhai et al. experimentally validate the similar phase accumulation zone that was attributed to the superior hardness of those regions to the local segregation of Ti [172]. The study depicted that the microhardness is marginally reduced by reducing the size of the sample. The obtained can be further examined in the other research studies depicting that the rate of solidification factor is directly related to the size of the sample. Therefore, the rate of solidification gets slow while reducing the size of the sample attributing to the rough microstructure, forming the softer material. The research outcomes revealed that microhardness is a variable factor depending on the processing parameters, the composition of alloy, powder quality, and heat treatment processes [145]. Even though the as-built material is harder than the conventionally processed material, it is still imperative to create innovative, custom heat treatment techniques that can effectively change the microstructure and harden the material even further. There is a theory that claims that because precipitation happens during processing, the material is already at its peak hardened condition and that any more heat treatment will cause it to age too quickly. In order to investigate precipitation behavior and, if necessary, define the sorts of precipitates that develop, atomic probe tomography may be useful.

#### 3.2.3. Tensile Characteristics of Laser-Based Additively Manufactured Al Alloy

**Figure 10:**
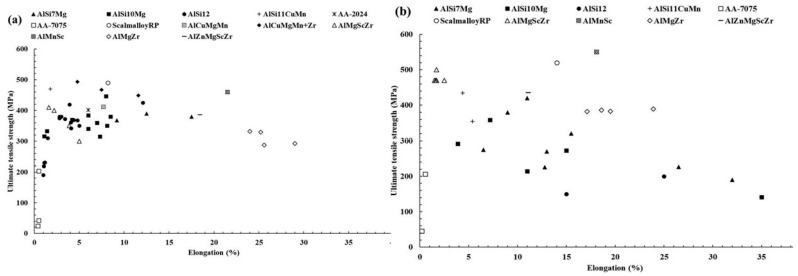
(**a**,**b**) Variation in the Tensile strength vs elongation (%) for Al-based material formed by SLM approach in as-built and heat-treated condition [145].

**Figure 11:**
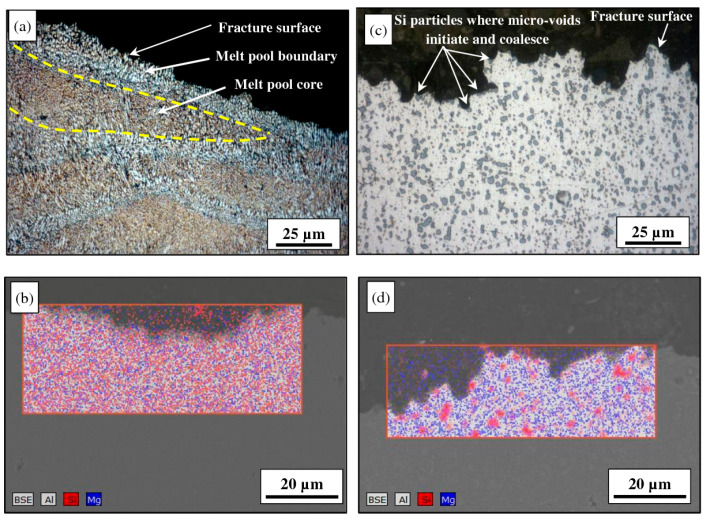
Tensile fracture of AlSi10Mg alloy formed by SLM approach oriented cross-sectionally comparing (**a**) as-built sample in the horizontal direction with EDS mapping (**c**), the heat-treated specimen (**b**,**d**). The fracture surface of an as-SLM specimen is oriented in the horizontal direction [161].

The fine microstructure of Al-based material obtained by the SLM approach attributing to the improvement in the tensile strength of the material. Figure 10 illustrates the variation in the tensile characteristics of various Al alloys highlighting the research findings. The formation of sub-grain and inter-dendritic barriers evolved the improvement in the tensile strength of the Al-based materials that prevent free movement of dislocation around the boundary [145]. The AlSi12 alloy is recommended for use in applications involving high-temperature conditions due to its small drop in tensile strength and marginal improvement in ductility under elevated temperatures [119]. Micro-cracking has been demonstrated to cause poor mechanical characteristics in high-strength alloys, as reported for AA-7075 [178]. Zr nanoparticles were used to functionalize the powder’s surface, which prevented cracking and improved the microstructure, giving the material tensile characteristics similar to those of wrought metal [179]. Although AA-2024’s strength and ductility were better than the non-heated cast samples, they were not as good as their aged wrought counterpart. Sc-containing alloys, such as Scalm alloy RP, produced ultimate tensile strengths of more than 530 MPa and elongation percentages as high as 14% as depicted in the research studies [145]. Other than that, the build orientation’s anisotropy had no impact on the tensile characteristics of the Al-based materials. The evolution of precipitation of the Al_3_Sc phase and super-saturation of Sc hinders the movement of dislocations around the boundary by pinning, attributing to the improvement in the tensile characteristics of the material [180]. The failure under tensile load surrounds the border of the molten pool when the material sample is aligned in a vertical direction, depicting the detachment of the fracture from the melt pool. The coarser grains or softer regions with fewer grain boundaries result in hindrance in the movement of dislocations [179]. The as-built specimens’ finer microstructure and more homogenous dispersion of the alloying components attributed to enhancement in the tensile characteristics of the Al alloy as shown in Figure 11a,c. The research outcomes depicted that Si gives the material the ability to strain harden which causes the cracks to start in the softer Al grains. As a result, the dependency on directional characteristics of Al alloy is controlled by the distribution of Si particles in the material [171]. When samples were oriented in the horizontal direction, the crack begins to propagate in the random direction spreading out in the melt pool. After heat treatment, cracks began to form and coalesce at the surface of Si particles, preferable for the crack initiation as shown in Figure 11b,d. Relating to the ductility of the materials, the spherical morphology of the Si particles serves the purpose of a stress concentrator that lowers the material ductility which is far superior as compared with the rod or needle-like morphology in the traditionally produced material [145]. Zr was incrementally added to Al-Cu-Mg-Mn alloys to increase their tensile characteristics and ductility [181,182,183,184,185,186] Additionally, Al-Mg alloys reinforced by Zr without Sc had exceptional tensile capabilities as a result of the development of Al_3_Zr precipitates (cuboidal), which were comparable to those of Sc-containing alloys without incurring the additional expense of adding Sc [145]. Al_3_Zr precipitates at nano- and sub-micron sizes helped to refine the grain, avoiding hot tearing in the solidification process and boosting strength via the Hall-Petch effect. In addition, the material has a far higher toughness than the alloys that contain Sc [187]. Resembling the low laser absorptivity of the consolidated metal, a shallower melt pool that was formed during the second scan enabled further refining. The research studies revealed that Zr-modified alloy had evolved higher ductility than the Sc-modified alloys. Changing the chemical composition of the Al-Si alloys is another way to significantly strengthen them while still utilizing SLM’s relative processing simplicity, as demonstrated by Pozdniakov et al. [145]. This alloy provided a compromise between strength and ductility through heat treatments.

#### 3.2.4. Compressive Characteristics of Laser-Based Additively Manufactured Al Alloy

Despite the improvement brought about by heat treatment, the compressive characteristics of the high-strength alloy AA-7075 were shown to be inferior as compared with the conventional counterpart. It’s important to remember that this finding applied to AA-7075 with the addition of 4% Si particles, attributing to the lessened cracking. The inability to restrict the propagation of cracks can lead to poor performance of AA-7075 alloy [145]. The compressive strength of an AlMg6.5 alloy with Sc and Zr additions was improved, and the results varied with the energy density [184]. The greater compressive strength (1.08 GPa) of the Al85Nd8Ni5Co2 alloy was maintained even at the higher temperature and corresponds to heat treatment [185,186]. The creation of a composite-like material after the SLM process in the form of an Al matrix supplemented with AlNd_3_, Al_4_CoNi_2_, and AlNdNi_4_; these reinforcements induced fracture deflection under stress [145]. Additionally, the distinctive microstructure produced compressive behavior that was superior to the counterpart that had undergone normal processing [186]. AlSi10Mg was reported to have a compressive yield strength three times greater than the cast material [145]. An AlSi12-TNM composite displayed increased compressive characteristics at the cost of flexibility attributed that the usage of reinforcement increasing the compressive strength of the alloy [145]. TiB_2_ particles were added to AlSi12 to increase its compressive strength while maintaining the material’s ductility by removing its crystallographic texture [187]. Although the compressive behavior of Al-based materials has not previously garnered much attention, it is now becoming more significant since aluminum alloys are utilized more frequently in lattice structures made by SLM, where compressive strength is the most critical mechanical characteristic. Overall, the research findings concluded that SLM-fabricated components have better compressive performance than conventionally-processed parts, making them appealing for a variety of applications.

#### 3.2.5. Fatigue Characteristics of Laser-Based Additively Manufactured Al Alloy

**Figure 12:**
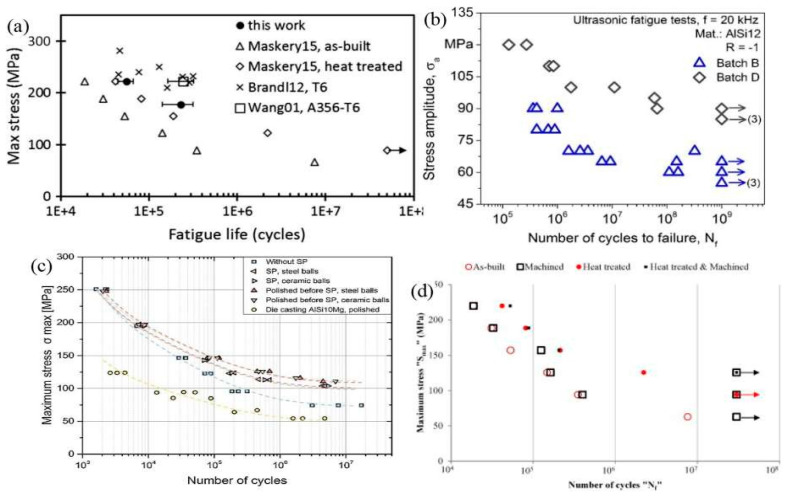
(**a**) fatigue characteristics for AlSi10Mg alloy formed by SLM approach, (**b**) S-N curves for AlSi12 alloy formed by SLM approach revealing the effect of build plate heating, where batch B does not involve build plate temperature while batch D involved build plate temperature of 200 °C, (**c**) S-N curves revealed shot peening of AlSi10Mg alloy sample, (**d**) S-N curves AlSi10Mg alloy revealed variation in fatigue characteristics corresponds to the machining of material and heat treatment [190,191,192].

**Figure 13:**
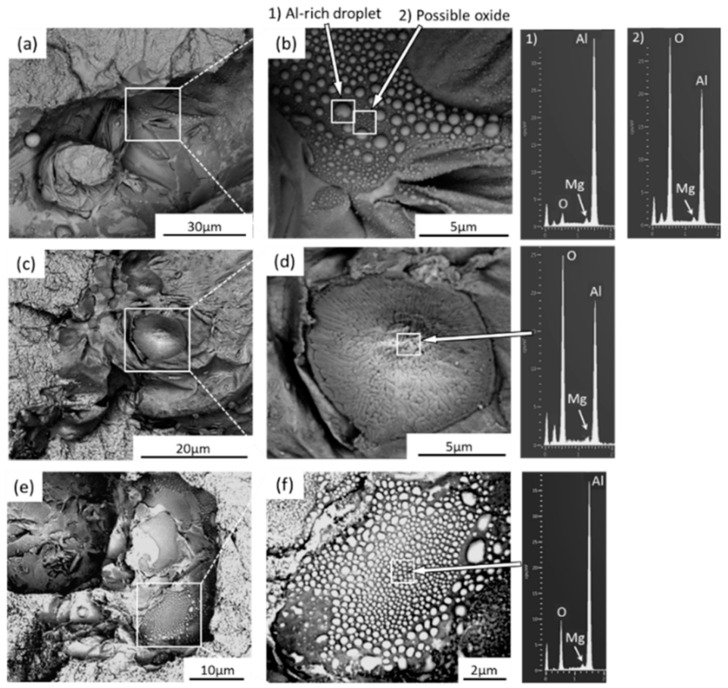
Backscattered electron images for oxide particles at the boundary between defect area and crack propagation region. An overview at lower magnification is given in (**a**,**c**,**e**), and detailed surface morphology in (**b**,**d**,**f**). Typical EDX spectra at right show the presence of Al and Mg in oxide particles [193].

SLM materials typically perform less well in terms of fatigue than their conventionally made counterparts. This has been linked to a number of causes in the literature, including the fact that second-phase particles in cast alloys make fatigue performance vulnerable to common SLM flaws including porosity, residual stresses, and surface roughness [145]. These serve as stress concentrations that shorten fatigue life by causing cracks to form and spread. The S-N curves from several studies are displayed in Figure 12, which contrasts the performance of the conventionally-cast material with the acceptable fatigue strength observed in SLM components made of Al alloys due to the finer microstructure created in the material [156]. A large difference in fatigue characteristics results from the variability between parts made from various powders on various systems utilizing a wide range of process settings. SLM parts made of alloys such as AlSi10Mg and Al6061, among others, have been reported to include internal pores harboring unbonded powder [161]. They may not significantly diminish the load-carrying area under tension while being relatively small, but during cyclic loading, they may have a more noticeable impact. The fatigue life is reduced as the flaw size increases [174]. Additionally, oxides that develop at these holes or are randomly distributed across the samples cause early failure. These oxides and holes are frequently seen where cracks first appear as depicted in Figure 13a–f. These oxides are thought to arise as a result of laser spatter, oxidized vapour, and the original oxide layer on the powder utilized in the process [188]. Supporting structures are added to sections with overhangs to increase the cooling rate, which forces the creation of a finer microstructure and increases the material’s fatigue strength. Brandl et al. observed that the build-plate temperature does not impact the fatigue strength of SLM AlSi10Mg but rather lowers data scatter [189]. The scatter in the results for AlSi12 alloy was also decreased by heating the build-plate. The improvement in fatigue strength contradicted a previous finding by Siddique et al., illustrated in Figure 12b. It’s unclear what caused this difference. Several techniques, including sandblasting, shot peening, rolling, burnishing, heat treatments, and hot isostatic pressing, can increase the fatigue life of parts. The fatigue strength of AlSi10Mg alloy was increased through shot peening employing steel and ceramic balls, outperforming samples that had undergone conventional processing as depicted in Figure 12c. According to Brandl et al., and Aboulkhair et al., a typical T6 heat treatment significantly increased performance, which is illustrated in Figure 12d by softening the material and therefore enhancing ductility [145]. The component is also vulnerable to premature failure when subjected to cyclic loads due to surface open pores or sub-surface porosity. A ring of porosity at the sample’s surface was seen by Damon et al. [145]. In the author’s work, it has been shown that milling, which is typically done to increase the surface roughness of SLM samples, causes sub-surface pores to come to the surface and increases data scatter. These pores were discovered to be the locations where cracks begin, spread, and ultimately fail. Yang et al., compared samples with machined surfaces that still had some sub-surface porosity to samples that allegedly had none. Both of them outlasted the life of the as-built samples, but the latter demonstrated a better fatigue life [176]. As a result, the sub-surface pores have a negative impact on the SLM samples’ fatigue life. Siddique et al. suggested repeatedly checking the shapes of the pieces for re-melting to lessen the likelihood of porosity development in these areas. Due to the direct relationship between a material’s ductility and fatigue strength, several process variables, such as build orientation and build-plate temperature, also have an impact on fatigue performance [121]. This was also anticipated for the material’s fatigue resistance, given that the ductility of the SLM material demonstrated signs of anisotropy dependent on the build orientation. The samples constructed in a horizontal position have a longer fatigue life than samples constructed in a vertical configuration.

#### 3.2.6. Fracture and Creep Characteristics of Laser-Based Additively Manufactured Al Alloy

The ability of a material to withstand crack propagation is determined by its fracture toughness. The ductility of a substance directly relates to its toughness. Despite having less ductility, the SLM material’s toughness significantly outperformed that of the cast material by a factor of almost three or four. This is caused by the interdendritic Si that is present at the Al cell borders and prevents the crack from spreading [145]. Additionally, the crack is forced to alter its course rather frequently as a result of the circular topology of the melt pools and the preferred crack propagation at their boundaries, which results in increased fracture toughness. As a result, samples formed in different build orientations have different fracture toughness with vertical samples having the lowest fracture toughness due to the anisotropic microstructure produced by SLM [165,166,167,168,169,170,171,172,173,174]. Annealing decreased the material’s fracture toughness even while its ductility and strength increased. The lower resistance to crack propagation, or poorer fracture toughness, is thought to be caused by the structural disintegration of the melt pool boundary. The heat-treated material was still twice as tough as its cast equivalent, though. SLM provides the opportunity to fabricate parts that simultaneously benefit from increased strength and ductility [20,40,41,42,43,44,45,145]. This advantageous mix of features cannot be obtained by utilizing standard processing methods. The evaluation of a material’s time-dependent mechanical performance at high temperatures is called creep resistance. It depends on the material when the temperature creeps become a design issue [145]. Al is understood to creep at temperatures between 200 and 300 °C because it has a comparatively low melting point [194]. According to Read et al., the creep resistance of SLM AlSi10Mg components is in line with expectations for this material, i.e., comparable to the material that has undergone conventional processing [195]. The creep resistance decreased as testing temperature or load/stress increased, as was to be predicted. At higher temperatures, recovery can begin, which causes creep resistance to degrade. Increasing the barriers that restrict the dislocation motion is one strategy for increasing creep resistance [145,174,195]. The research studies depicted that the high dislocation density of Al alloy formed by the SLM approach attributed to the improvement in the creep resistance of the alloy. It is anticipated that these dislocations will entangle and function as barriers to one another, increasing the resistance to deformation. In contrast to the tensile characteristics, creep resistance is typically improved for materials with bigger grain sizes. This is because the higher grain size reduces the diffusion rate and restricts the sliding of grain borders, two factors that are crucial for creep. For age-hardenable Al alloys, the presence of precipitates in the material also increases creep resistance [196].

#### 3.2.7. Impact and Wear Resistance Laser-Based Additively Manufactured Al Alloy

Analyzing the impact resistance behavior is likely more pertinent for lattice structures in the SLM sector. This is caused by their potential for usage in energy absorption applications and as an impact-protective mechanism. The most common method of evaluating a material’s impact resistance is to experimentally measure the impact energy, or the effort required to shatter a specimen. After impact, the sample absorbs the energy up to the yield point, at which point plastic deformation begins [140,141,142,143,144,145]. The material breaks as it hits the limit and is no longer able to absorb additional energy. The complex dynamic behavior of the material can be controlled by regulating the struggle strain hardening developed in the material (to peak flow stress) followed by thermal softening. SLM parts outperformed their traditionally processed counterparts in terms of impact resistance, similar to the other sorts of mechanical strengths that have been discussed up to this point [190]. Although this was not anticipated because poor impact resistance often goes hand in hand with low ductility. As-processed SLM samples from Charpy impact testing had impact energies that were either on par with or superior to (by about 1.5 times) the cast material. The dynamic yield strength of heat-treated samples from planar impact testing was nearly twice as high as the dynamic tensile strength of the sand-cast counterpart [176]. The finer, more homogenous microstructure is what gives the material its increased impact strength. Impact resistance was not affected by anisotropy, despite it having considerable influence on some mechanical parameters (the material’s resistance to impact was independent of the build orientation) [197].

**Figure 14:**
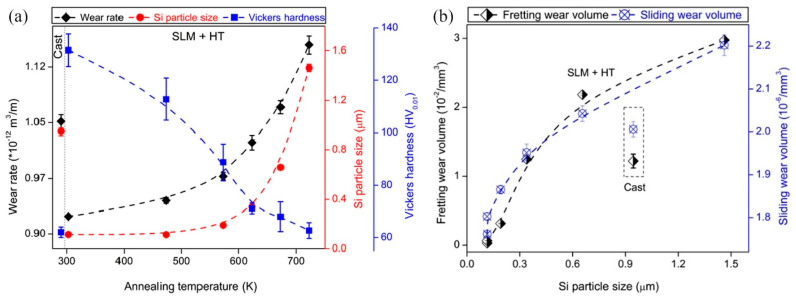
(**a**) Influence of heat treatment process on the hardness and wear of AlSi12 alloy formed by SLM approach, (**b**) tribological behavior of the materials formed by SLM approach comparable to cast counterpart [145].

For the advancement in the automobile sector where a part may undergo a large degree of friction in an application, the tribological behavior of an SLM Al part is very crucial [145,198]. In the family of Al-Si alloys, the wear resistance of the material increases with increasing Si concentration [191]. SLM’s finer grain structure, which outperforms that of its cast and extruded counterparts, results in high resistance to wear in the sliding wear condition. For wear caused by corrosion and erosive processes, similar outcomes were seen [199,200]. The materials can be strengthened to increase their wear resistance [145]. In comparison to ceramic reinforcements, metallic reinforcements offer superior compatibility with the parent metal. The SLM process settings have an impact on the wear mechanism, which is primarily abrasive for higher-hardness materials and shifts to the adhesive as the hardness declines. The hardness of a substance directly relates to how resistant it is to wear. As a result, parts in their as-built condition show the lowest wear rate, whereas annealing softens the material and increases the wear rate, as shown in Figure 14a,b which compares the SLM material’s tribological behavior to that of its cast equivalent [201,202]. The greater tribological behavior is warranted because SLM procedures result in material that is tougher than what is produced by conventional processes. The presence of a surface oxide layer, which is eliminated during the initial rounds of sliding in a wear test, causes the coefficient of friction in SLM samples to be greater at the surface [203]. Beyond this layer, the coefficient of friction stabilizes and starts to reflect the capabilities of the material more accurately. This was attributed by Kang et al. to the weld-fracture process that was prevalent in soft metals prior to stabilization [145]. Variations in the coefficient of friction patterns are one way that this is seen. The softer the material, the longer the fluctuation region [204,205]. Due to the limited weld-fraction process, samples with substantial porosity typically don’t exhibit any fluctuation region.

### 3.3. Metallurgy of Laser-Based Additively Manufactured Al Alloy

#### 3.3.1. Microstructure of Al Alloy Formed by SLM

**Figure 15:**
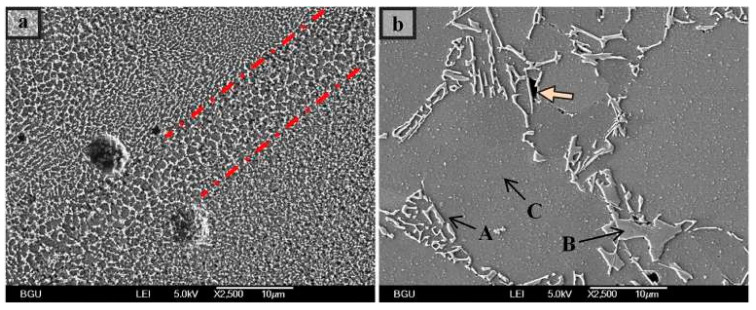
SEM image depicted AlSi10Mg microstructure formed by (**a**) SLM, and (**b**) casting. The arrows in (**b**) point to (A) Al-Si eutectic, (B) Si dispersed in Al matrix, and (C) Fe-containing intermetallic phases [191].

**Figure 16:**
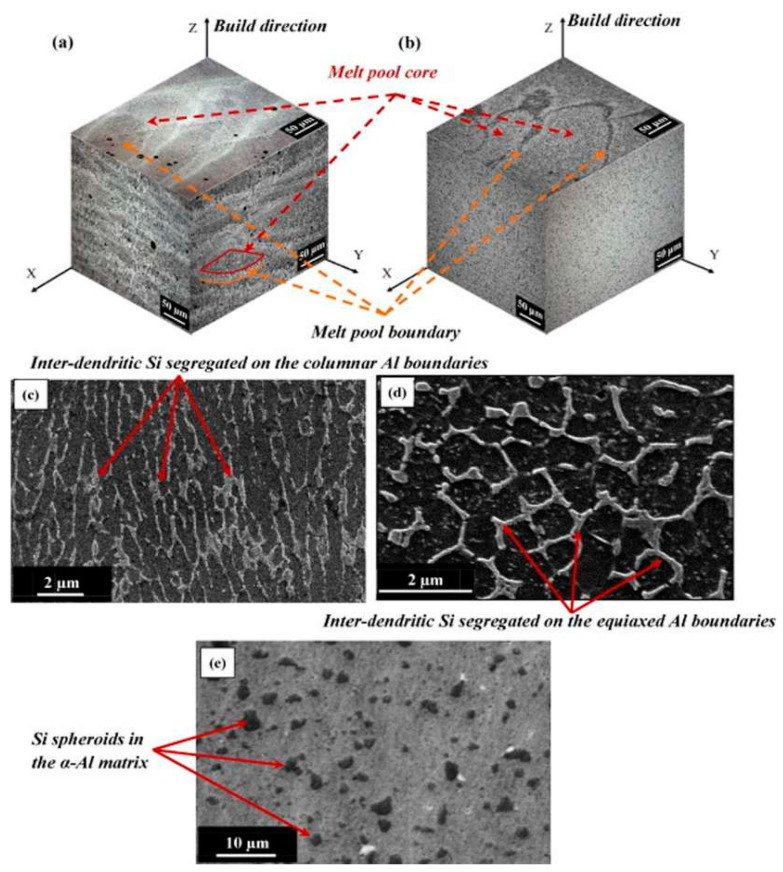
The microstructure of SLM AlSi10Mg is shown in isometric views in the following order: (**a**) as-built, (**b**) after heat treatment; (**c**,**d**) elongated α-Al and equiaxed α-Al grains as seen on the XY plane in the as-built material and (**e**) Si spheroids in the α -Al matrix after T6 heat treatment [192].

**Figure 17:**
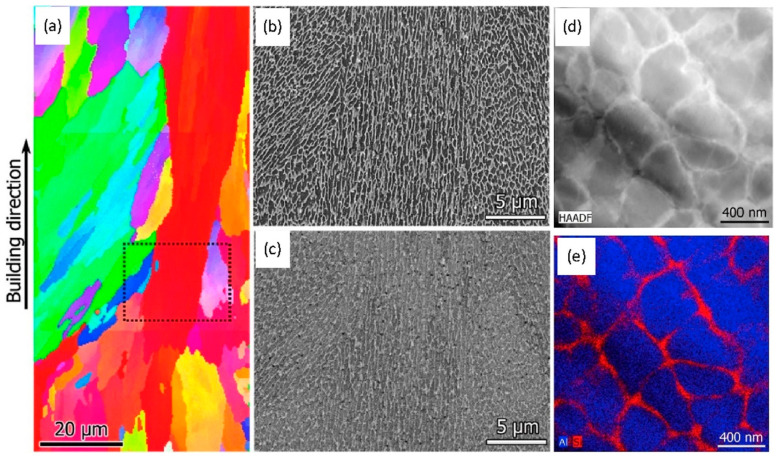
The (**a**) EBSD image depicts the AlSi10Mg alloy grain structure obtained by SLM with columnar cells developing perpendicular to the build direction, (**b**,**c**) SEM images show the microstructure in the dashed region and the grain structure using a secondary electron detector, and a backscatter electron detector, respectively. As-built SLM AlSi10Mg cells’ STEM images and associated Al-Si EDX maps are displayed in (**d**,**e**) [207].

The creation of the microstructure is governed by the temperature during SLM. During processing, the material is exposed to directional heat transfer and significant thermal gradients [145]. It is also repeatedly remelted as a result of internal heat transfer and the laser beam’s ability to pass through layers. With increasing laser power and scan speed, solidification happens at an incredibly fast pace (103–108 K/s), resulting in a thin microstructure with metastable phases [156]. As an alternative to the coarse microstructures created by normal manufacturing, this fine microstructure is in demand. In Figure 15a, the comparison of the microstructures created by casting and SLM is indicated. The microstructure outlined above was discovered to be in conformity with the sequence provided by the AlSi10Mg phase diagram estimated using Calphad by Takata et al. [201]. They also noted that the size of the generated sample affected the microstructure of the material created. Si particles were found inside the columnar Al grains of smaller samples, ranging in size from 0.1 to 0.3 mm, demonstrating that Si precipitated during SLM under these circumstances [145]. The effectiveness of the heat flow can be used to explain this. When compared with the solidified material surrounding the melt pool in the case of the bigger samples, the melt pool in the smaller samples is surrounded by unmolten powder, which has a reduced heat conductivity. Due to the comparatively modest rate of solidification imposed by the decreased thermal conductivity and the lengthy periods of increased temperatures, Si can precipitate in the columnar Al grains [206]. Figure 16b depicted the microstructure of SLM AlSi10Mg in isometric views for as-built and after-heat treatment. Figure 17 entails the EBSD image of the AlSi10Mg grain structure produced by SLM with columnar cells growing perpendicular to the build direction, and SEM images show the microstructure in the dashed region and the grain structure using a secondary electron detector and a backscatter electron detector [207].

#### 3.3.2. Crystallographic Texture of Laser-Based Additively Manufactured Al Alloy

**Figure 18:**
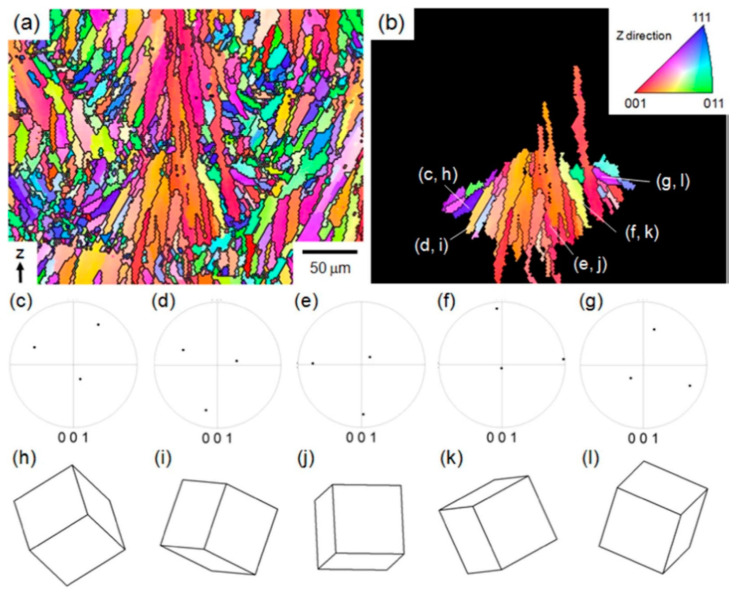
(**a–l**) Inverse pole figure orientation map displaying the elongated grain structure’s predominately {1 0 0} orientation along the build direction. Additionally, the orientation map reveals a finer grain structure at the melt’s sides and top, but there is no clear dominating orientation [208,211].

Despite the lack of mechanical processing necessary for deformation texture in SLM, temperature gradients and rapid cooling rates encourage the epitaxial development of columnar grains in the majority of Al alloys. This texture results in mechanical anisotropy, crack susceptibility, yield strength, and elongation at failure. The directional solidification within the melt pool is the source of the crystallographic texture found in SLM Al components [145]. However, the selection of processing settings and the thermo-physical characteristics of the material have a considerable impact on the geometry of the melt pool, which in turn affects the heat flow direction at the liquid/solid boundary and the pace of solidification [176]. As a result, depending on the tools and feedstocks employed, the strength of the final grain texture varies greatly. The average grain growth direction during the initial phases of melt pool solidification depends on the solidification front direction (usually perpendicular to the melt pool boundary) and thermal gradients. Due to constitutional undercooling being prevented in the majority of Al alloys due to high thermal conductivity and solidification velocities, thermal gradients are primarily in the opposite direction of the build direction, or radial, depending on the melt pool width-to-depth ratio [145]. These circumstances produce morphological grain texture, with elongated grain structures emerging from the melt pool boundaries in the longitudinal cross-section of SLM components. Research has in fact proven the relationship between the morphological and crystallographic textures of the grain created by the melt pool boundary. According to Wu et al., elongated grains of SLM AlSi10Mg (discovered close to melt pool boundaries) are made up of sub-cells with the same orientation. According to Thijs et al. [140], this dominant grain orientation results in a fiber texture component with a {1 0 0} along the scan direction depicted in Figure 18. Figure 18 also depicted the Inverse pole figure orientation map displaying the elongated grain structure’s predominately {1 0 0} orientation along the build direction. Additionally, the orientation map reveals a finer grain structure at the melt’s sides and top, but there is no clear dominating orientation [208]. Suryawanshi et al. reported similar outcomes for AlSi12 alloy as well. The elongated grain structure of AlSi10Mg and Al-Mg-Cu, on the other hand, has a dominant {1 0 0} texture along the build direction, as shown by Takata et al. and Zhang et al. [208,209,210,211,212,213,214]. These variances are attributed to variations in the melt pool shape geometry and consequently thermal gradients, while not being stated directly. Elongated grains either consume the residual liquid when the melt pool solidifies or a refined equiaxed grain structure develops [156]. Equiaxed grains are those that have no prominent crystallographic texture and are desired to minimize mechanical anisotropy. They are anticipated to form in alloys with narrow solidification ranges from surface nucleation. Recent studies have concentrated on methods to create a melt pool with a more refined, homogeneous structure that would eliminate any crystallographic roughness and lessen solidification cracking. It has been proven successful in promoting refined texture-free melt pool grain structures in a number of Al alloys, including Al-Mg-Zr, AA-2XXX, AA-6061, and AA-7075, by adding suitable heterogeneous elements that increase the density of nucleation sites in the melt pool and encourage columnar to the equiaxed transition of the grain structure [145].

### 3.4. Precipitation Hardening of Aluminum-Based Alloys

Precipitation hardening has relied on solid-state transformations, which are carried on by a reduction in one or more alloying elements’ solid solubility with a rise in temperature [215]. This makes it possible for the alloying components to dissolve while being held at high temperatures. After quick quenching, an out-of-equilibrium SSSS is produced, which, when held at room temperature (for natural aging) or elevated temperatures (for artificial aging), decomposes via diffusion and results in the controlled generation of finely dispersed precipitates. Multiple intermediary steps are taken in the decomposition of the SSSS to reduce the amount of activation energy needed. The process normally starts with the formation of small, coherent clusters of solute atoms, which subsequently elastically strain the matrix around them to strengthen the alloy. From there, larger than the initial clusters intermediate precipitates form that have a consistent, though variable, composition and crystal structure. Finally, a stable precipitate is created, which is typically less effective at strengthening the alloy because it is bulky and less coherent when compared with the matrix. Chemical hardening (i.e., resistance to shearing by dislocations), lattice distortion, and dispersion hardening (i.e., Orowan strengthening) are the three fundamental factors that lead to precipitation hardening [216,217].

#### 3.4.1. Al-Si-Mg Alloy System

Alloys based on the Al-Si system are part of families that are optimized for both casting (such as A357-AlSi7Mg) and plastic deformation. Mg is frequently added to these alloys to produce precipitation hardening (i.e., the AA6000 series). The two families’ precipitation sequences, which are based on the face-centered cubic Mg_2_Si phase, are very similar:

SSSS → Mg/Si atom clusters → GP zones → β″ (coherent needles) → β′ (semi-coherent rods/laths) → β (incoherent platelets)

Another potential phase that has been mentioned is the B′ precipitate (Al3Mg9Si7). Numerous studies have shown that the quantity of retained vacancies, which may also be impacted by the quenching rate and potential pre-aging treatments, is a factor in the development of solute clusters and GP zones [218,219,220,221]. Thermal treatments can also cause the precipitation of dissolved Si [222,223] or a change in the shape of the eutectic Si, as reported in [224,225], in addition to the creation of the Mg_2_Si phase. It has been claimed that Si interdiffusion, rather than surface diffusion, is likely to be the cause of the latter process.

#### 3.4.2. Al-Zn-Mg Alloy System

The most significant Al-Zn-Mg-based alloys are those from the 7xxx series of wrought alloys; these alloys are well known for their superior mechanical qualities and positive response to age hardening. This system’s precipitation pattern is based on the hexagonal MgZn_2_ phase:

SSSS → GP zones → η′ (semi-coherent disks) → η (incoherent)

Vacancy-solute clusters have been suggested to have a function in the early phases of aging in contrast to the establishment of GP zones [226]. If aging is carried out at a high temperature, the production of an incoherent cubic T phase with a composition similar to Mg_3_Zn_3_Al_2_ phase may occur [227]. Furthermore, it has been demonstrated that high dislocation densities prevent the growth of GP zones and clusters (likely by eliminating vacancies) while simultaneously encouraging the precipitation of η by acting as nucleation sites [228].

#### 3.4.3. Al-Mg-Sc-Zr Alloy System

The addition of Sc and Zr to Al-Mg alloys from the 5xxx series has been the subject of much research in recent years. Sc is added, and this results in various advantages. For instance, initial Al3Sc particles with an L12 crystal structure are created during solidification and, due to their low lattice misfit to Al, serve as heterogeneous nucleation sites. In turn, this causes general grain refining and decreases solidification cracking. Additionally, as the alloys age, secondary Al3Sc precipitates may also form, enhancing the mechanical resistance of the alloys. Such precipitates have an extremely low lattice misfit (δ = 1.33%) and are coherent with the aluminum matrix. The critical radius for the coherent to the semi-coherent transition of Al3Sc precipitates is 20 nm in theory, but coherency is actually maintained by larger particles, primarily due to the presence of Mg in the aluminum matrix [229]. This coherency is maintained even after annealing at relatively high temperatures for a prolonged period of time (e.g., 300 °C to 450 °C for 168 h) [230]. Significant attempts have been made to increase the solid solubility through quick solidification [231] since Sc has a low solid solubility in the aluminum matrix under equilibrium conditions (0.38 wt.% at 660 °C [230]), which weakens the precipitation. The high cooling rates typical of LPBF are therefore predicted to extend the solid solubility of Sc and may provide an additional advantage if aging treatments are to be used, making this family of alloys extremely interesting candidates for the LPBF process [232]. Additionally, it has been demonstrated that larger concentrations of dissolved Sc improve the kinetics of the precipitation process, indicating that at least somewhat faster precipitation could be anticipated in LPBF alloys [231]. Al_3_(Sc, Zr) precipitates are created when Zr is added to the alloy, and they have a core-shell structure [233] and great thermal stability [234].

### 3.5. Heat Treatment of Laser-Based Additively Manufactured Al Alloy

**Figure 19:**
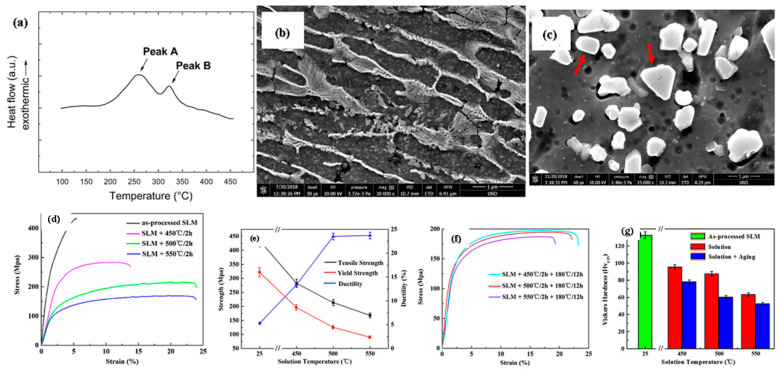
(**a**) DSC thermogram of an LPBF AlSi10Mg alloy, (**b**,**c**) SEM micrographs of as-built and solution-treated and quenched LPBF AlSi10Mg alloy where arrow represents that After solution treatment, it is possible to see how the eutectic network vanishes and how Si particles become more coarse), and (**d**–**g**) Mechanical characteristics of solution treated AlSi10Mg alloy [236].

Since Si-containing alloys were the subject of the majority of the earliest research on LPBF of aluminum alloys, these alloys have also been the main focus of efforts to find suitable heat treatments [145]. This research has mostly focused on three alloys: the hyper eutectic AlSi12 alloy, the hypoeutectic, age-hardenable AlSi7Mg alloy, and the AlSi10Mg alloy. Research has been put into creating new annealing techniques that can enhance the mechanical (strength, ductility, fatigue resistance) and functional properties (corrosion resistance, thermal, and electrical conductivity) of the alloys ever since the significance of the post-production heat treatment of LPBF aluminum parts was first realized [171]. First, to improve the characteristics of LPBF aluminum alloys, typical thermal treatments (such as T6) were modified in terms of temperature and duration (e.g., T6). Secondly, annealing treatments at various temperatures were investigated to comprehend the caused phase transitions [235]. As a result, direct aging (T5) and customized annealing procedures were created, taking into account the LPBF Al-Si alloys’ unique microstructures. In particular, a number of investigations have been conducted utilizing differential scanning calorimetry (DSC) to characterize the phase transitions that occur when heating Al-Si-Mg alloy [236]. To learn the metallurgy of the alloys and their response to heat treatments, one essential first step is to comprehend such transformations. The thermograms that were recorded and displayed in Figure 19a are distinguished by the existence of two exothermic peaks: peak A, which is located at about 260 °C, and peak B, which is located at 320 °C [237]. According to many authors’ interpretations, the cause of the existence of these peaks has been extensively explored and supported in various ways. Peak B was assigned to the superposition of two effects (i.e., the precipitation of β′ and the rupture and spheroidization of the Si network), according to Fiocchi et al. [238]. Peak A was attributed to the precipitation of the Mg_2_Si phase in its coherent β” form. Yang et al. [239] attributed peak A to β″ precipitation and peak B to β′ (attributed to the collapse of cellular Si walls). Marola et al. [240] attributed peak A to the precipitation of Si from the supersaturated aluminum matrix and peak B to the concomitant production of Mg_2_Si and Fe-containing precipitates based on their respective enthalpies. Similar conclusions were reached in [241] and strengthened by the observation of a single exothermic peak in a binary Al-50Si alloy between 196 and 299 °C [109]. Peak B exhibits characteristics of both Si diffusion and β’ peak creation, hence Girelli et al. [237] came to the conclusion that neither phenomenon can be attributed to peak B with certainty. Table 3 identified the effects of heat treatment on the Al alloys. With cooling rates of 105–106 K/s, a material processed by SLM solidifies in microstructures that are distinctively different from those attained through conventional processing, which uses lower cooling rates [145]. The size of the grain structure is the primary distinction between SLM materials and those that have undergone conventional processing. However, this will also rely on the material being treated, based on the response of its constituent elements to laser irradiation. Conventional processing often results in coarse microstructures, whereas SLM creates far finer microstructures [145].

The process started with a high-temperature solution annealing step. Prior precipitates and intermetallic phases are intended to be dissolved, and quenching is then used to create an out-of-equilibrium SSSS. The SSSS is then annealed at a relatively low temperature, typically between 150 and 250 °C, which causes the precipitates to develop in a finely dispersed state and reinforce the aluminum matrix [236]. The T6 term, which is frequently used, specifically refers to the condition of maximal strength reached [145,236]. As depicted in Figure 19b,c, Solution and aging treatments significantly alter the microstructure’s appearance and characteristic dimension, which has a profound impact on the treated alloys’ mechanical and functional characteristics [236]. Less obvious but no less significant changes in the distribution of other elements and the production and dissolving of precipitates also take place during solution heat treatment. Even following high-temperature solution treatment, the existence of nm-sized Si precipitates, which are typical of as-built samples, within the α-Al matrix has been demonstrated [246]. In addition, solution treatment has been frequently documented to cause the needle-like monoclinic β-Al_5_FeSi phase to develop. During subsequent high-temperature holding, Fe diffuses to create Al5FeSi, which is thought to be embrittling and therefore harmful to the alloy’s mechanical behavior [236]. In the as-built condition, Fe segregates at the cell and grain boundaries, finally creating the π- Al_8_Si_6_Mg_3_Fe phase.

Contrarily, solution treatment had little to no impact on grain size, with the bulk of columnar grains mostly remaining constant, notwithstanding a reported minor expansion of small equiaxed grains at melt pool margins [246]. As a result, heat treatment has no effect on the type or degree of texture. Regarding the impact of solution treatment on residual stresses, there is no entire evidence identified. After solution treatment and quenching, the investigated alloys are exposed to artificial aging; however, no significant changes in the microstructure are carried about on a wide scale (i.e., in Si morphology and grain size) [236]. However, as anticipated, the reinforcing Mg_2_Si phase precipitates. There are disagreements on the precise order of precipitation and whether the B’ precipitate (Al3Mg9Si7) should also be taken into account, despite the fact that a similar precipitation sequence has been described for the comparable cast Al-Si-Mg alloy. Microstructural alterations have a strict influence on how mechanical characteristics evolve during solution treatment [246]. All the literature that was studied noted a reduction in strength following solution treatment, which intensifies with an increase in treatment temperature or time, as seen in Figure 19d–g [236].

Other than those based on Al-Si systems, the variety of scientific publications addressing heat treatment of LPBF aluminum alloys is considerably less. Due to the wide solidification range and solidification shrinkage, making such alloys has actually been considerably more challenging [145]. As a result, widespread cracking has been documented in high-strength alloys such Al-Zn-Mg and Al-Cu alloys [236]. Numerous studies have looked into altering these alloys by adding either additional alloying elements (like Si) or in situ and ex situ inoculants (e.g., TiB_2_ or SiC nanoparticles) in order to solve these processing problems [246]. Particular attention must be taken while studying these modified alloys in view of the optimization of heat treatments, since their as-built microstructure and precipitation sequences may be significantly changed. Despite significant changes in the as-built microstructures (grain size and shape, dislocation density, etc.), TiB_2_ inoculants were demonstrated to have no effect on the kinetics of precipitation in Cu-based 2618 (Al-3,5Cu-1,5Mg-1Si) alloys [236]. On the other hand, the addition of Si to an Al-Zn-Mg 7075 alloy caused the Mg_2_Si precipitation pattern to develop in addition to the typical Mg_2_Zn sequence [221]. In order to fully use the strength potential of various alloys, investigations have concentrated on the heat treatment of such materials.

Ostwald ripening is a phenomenon that occurs in solid solutions or liquid sols and refers to the gradual transformation of an inhomogeneous structure, in which smaller crystals or sol particles dissolve and then reappear on bigger crystals or sol particles [211]. The large particles will often expand while the little particles tend to contract [211]. As a result, the dispersion of sizes will reduce and the average size of the nanoparticles in the solution will increase. Since the research study showed that the Si atoms in the AlSi10Mg alloy were pushed in front of the solid-liquid barrier, this increased the concentration of Si atoms surrounding the main α-Al in the liquid phase [212]. The continuous network eutectic structure (α -Al + Si) thereafter came into being. Furthermore, at a heat treatment temperature of 225 °C, the network eutectic Si started to disintegrate and grow. The prior continuous network Si structure was replaced by the coarse Si particles, which were disseminated throughout the network structure as the heat treatment temperature rose from 225 °C to 275 °C. The eutectic Si entirely changed from network particles to block-like and spherical particles at the final heat treatment temperature of 325 °C [212].

### 3.6. Feedstock: Pre-Alloyed Powder and In-Situ Alloying

Researchers who process Al using SLM have paid the most attention to the usage of conventional pre-alloyed powder; this may be because they are widely used in other industrial processes [145]. Additionally, the majority of current research aims to construct a part that is already made traditionally using SLM without modifying the material. AlSi10Mg and AlSi12 alloys have undergone significantly more work than any other alloy [201]. The SLM powder specifications are taken into account according to the process requirements. In order to accommodate the requirement for consecutive deposition of homogenous powder layers, the powder should have a spherical morphology [236]. This morphology is necessary to improve packing density and flowability. The beginning powder contains gas phases that significantly reduce densification. These may be internal gas pores that are trapped during the powder production process, or they may be surface pores caused by moisture, which aids in the generation of hydrogen porosity [236]. It is also advised to pre-heat the powder before processing in order to increase its absorptivity. In SLM, metal powders are either gas-atomized or plasma-atomized, with the former being more prevalent. According to the powder’s manufacturer, powders from the same alloy might also have different morphological characteristics. 

In-situ alloying is a promising method for creating unique alloy compositions that will improve both the characteristics of the parts and the SLM process-ability [236]. Al alloys could be coated with substances or mixed with substances that would increase their absorptivity or surface tension, for example, to improve the material’s processing capacity. Particles with intermediate absorptivity and reflectance as compared with the original elements are produced by depositing Cu on Al particles [176]. The perspective of the SLM process may change as a result of in-situ alloying because it has the potential to significantly increase the material options available to the technology. Understanding how various materials react to laser irradiation during processing, both individually and in combination with other materials, is one of the challenges in designing alloys in this way, as this is necessary to enable the prediction of the characteristics and properties of the resulting material. Ti, Al, and Nb were combined in a turbula mixer by Grigoriev et al. [218] to form Ti_2_AlNb components with a largely homogenous elemental distribution, albeit some partially molten Nb particles were also scattered unevenly. The ability to modify the composition of a commercially available alloy by adding specific additives driven by different driving forces is another benefit of in-situ alloying. Al’s microstructure was improved by Bartkowiak et al. [210] by the addition of traces of Zn and Cu. By using TiB_2_ particles, Xi et al. [180] were able to remove the crystallographic texture from the AlSi12 alloy. AlSi18 alloy was created by Kang et al. [183] using a tumble mixer with AlSi12 and pure Si particles. Starting with Al4.5Cu and gradually adding increments of Cu, Wang et al. [211] drum hoop mixer produced a variety of Al-xCu alloys in-situ improving the material’s compressive strength at the expense of its ductility. SLM has also been utilized to generate reinforced Al-based metal matrix composites (MMC) in-situ, with the reinforcements dispersed throughout the material to give results that are superior to those of traditionally processed composites [211]. AlSi10Mg and SiC were used to create innovative Al-based composites by Gu et al. [166], which had numerous reinforcements and improved mechanical properties. Zhao et al. [236] demonstrated three types of reinforcements—unmelted SiC, Al4SiC4, and the eutectic Si phase—form in SiC/AlSi10Mg composites. Carbon nanotubes (CNT) were added to AlSi10Mg by Wang et al. [251] using ball milling, and the composite was then treated by SLM. The inability of x-ray diffraction to detect the existence of aluminum carbides in the material led to the belief that the laser energy destroyed the CNT’s distinctive structure and caused the carbon to evaporate.

### 3.7. Correlation with Grain Orientation, Grain Size, and Aspect Ratio

**Figure 20:**
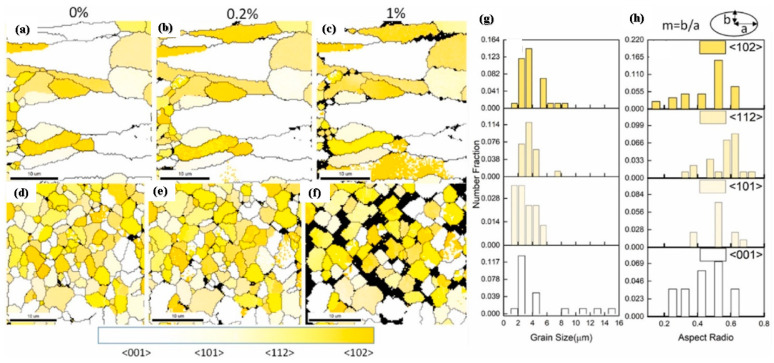
(**a**–**f**) Mapping distribution of grain orientation, (**g**) grain size, and (**h**) grain aspect ratio of SLM Al–Mn-Sc alloys [252].

Although the initial grain orientation, grain shape, and grain size were connected to the lattice rotation, a quantitative expression was missing. Figure 20 displays distribution maps of grain sizes and shapes at various strain levels as well as the four different types of grain orientation maps [252]. The grain shape is represented by the aspect ratio (m). The grain size of the remaining orientations was primarily distributed between 1 and 6μm, with the largest grain size dispersed in grain orientation <001> [252]. All grains’ aspect ratios fall primarily between 0.2 and 0.6. According to the analysis of the rotation angle research study [252], the 001 orientation has the largest rotation angle because dislocation slip can be activated easily, which is not good for increasing strength. In contrast, the <102> and <101> orientations have smaller rotation angles because dislocation slip can be activated more difficultly, which can effectively increase strength. The lattice rotation, which shows the anisotropy of the mechanical characteristics in the longitudinal and cross-sections, is sometimes influenced by the grain orientation, size, and aspect ratio [253,254,255]. Since, the rotation angle fraction is expressed as the ratio of the remaining rotation angles to the greatest rotation angle, where the maximum rotation angle is defined as one in order to normalize all described grains. Prior to investigating their impact on the rotation angle caused by dislocation slip, the grain size and aspect ratio must be known. The rotation angle fraction is expressed in the studies as a function of grain size and grain aspect ratio. According to Li et al. [256], the microstructure of AlSi10Mg alloy produced additively and with various grain sizes and shapes displayed blatant mechanical property anisotropy. The impact of grain form and crystallographic texture on the mechanical properties of high-strength aluminum alloys was demonstrated by Romanova et al. [257]. Winther et al. [258] demonstrated that the primary factor governing the lattice rotation was the initial grain orientation.

### 3.8. Challenges in Laser-Based Additively Manufactured Aluminum Alloy

The demand for sacrificial support material to provide mechanical fixturing for the part as it is being created is one of the most important difficulties with SLM design. For vast overhang regions, downward-facing surfaces, or disconnected portions, they are necessary [156]. Importantly, they also offer the main method of preventing part distortion during fabrication as a result of residual stresses brought on by the significant temperature differences encountered during the process. The inclusion and positioning of features such as holes and internal channels in designs for SLM are constrained by this necessity. Because adding support to designs requires human pre-processing, support structures also raise manufacturing costs [156]. Additional time is required to create support structures, post-processing, and support removal and surface polishing to remove artifacts and waste material from an SLM perspective, but it can be scrapped and re-melted for other uses [175]. Parts should ideally be modified to become self-supporting. However, focusing optimization primarily on lowering the utilization of support structures may have unfavorable consequences for the part’s functionality [145]. The cost of support material and the overall effects on the design should therefore be carefully balanced by the designer. The orientation of the part in the machine must also be taken into account in designs as it may have an impact on a number of variables, including production time, the need for support structures, residual stresses, surface roughness, microstructure, and the impacts of build anisotropy [236]. In the future, it may be possible to incorporate SLM production limitations, which would lessen the amount of manual work needed by designers to select the appropriate orientation, eliminating the need for support structures, and increasing part quality. There are two ways to look at the mechanical characteristics of SLM parts [198]. The first is structural integrity, where SLM parts may have flaws such as surface imperfections, porosity, fissures, inclusions (such as trapped laser splatter), and excessive residual stresses. A combination of material specification and post-processing treatments are frequently required to repair such faults, allowing the fabrication of fully-dense defect-free parts. The existence of such defects can drastically decrease mechanical performance [236]. The second viewpoint is in the field of metallurgy, where the more refined microstructures created by SLM materials might improve particular mechanical characteristics for diverse materials systems. Depending on the build orientation and scan approach employed, SLM parts can also display anisotropic mechanical behavior.

## 4. Conclusions and Future Prospects

The work-study successfully examines the advancement of magnesium and aluminum alloy in the role of additive manufacturing. The laser-based additive manufacturing approach is a quite useful technique used for as-cast and heat-treated magnesium and aluminum alloy. The investigation of various aluminum and magnesium alloy is successfully accomplished. The influence of laser-based additive manufacturing on the mechanical characteristics of magnesium and aluminum alloy is examined. The advancement of additive manufacturing in building light-weight materials (magnesium and aluminum material) has been reviewed. The investigation of novel magnesium alloys has a lot of potential components that are incredibly light and use empty space as a design factor. As with any new development in this environment, it would broaden the usage base as the qualities of the materials change to meet the needs of new markets. New smart devices and components for biomedical applications can suitably be formed using the LBPF approach of additive manufacturing for Mg-based alloys. Today, a sizable amount of work is being done in the area of SLM of Al alloys. To focus future research on areas that can raise the status of the technology, some restraint is required. There is currently a dearth of research into how the sample size affects the choice of process parameters. It has not yet been thoroughly investigated how these process parameters translate to the manufacturing of noticeably small features, such as in the case of lattice systems. Custom scan strategies for lattice structures can raise their performance by raising their quality. Two paths have been taken in research to address the flaws in SLM parts: in-process or post-processing. Higher build-plate temperatures have the ability to reduce the flaws formed throughout the process, but they may have an adverse influence on the material’s microstructure, mechanical characteristics, and process efficiency. For the process to achieve its demands by creating powder with specifications meeting the process criteria, it is crucial to standardize the qualities of metal powders for SLM. Despite the fact that powders that don’t completely meet the standards for the process can still be used to produce parts without any defects. To fully utilize this potential, high-throughput methods to experimentally evaluate the bespoke alloys and materials design software are required, as well as understanding how the alloying components affect the material’s capacity to be processed by SLM and, eventually, the qualities of the manufactured parts in use.

## Figures and Tables

**Table 1:** Processing parameters of Mg-based alloy prepared by Additive Manufacturing powder-based approach.

Alloy Components	Size (μm) and Shape of Powder	Methodology	Parameters Used in Powder-Based Additive Manufacturing Approach					Input Energy Density (J/mm^3^)	Relative Density (%)	References
			Power (W)	Spot Size (μm)	Speed (mm/s)	Layer Thickness (μm)	Hatch Spacing (μm)			
Mg (Pure)	Pre-alloyed 24 μm, spherical shape	Laser-powder bed fusion	75	85	500	25	35	155	96.5	[44]
					1240			63	88.2	
Mg (Pure)	Pre-alloyed 43 μm, spherical shape	Laser-powder bed fusion	85	90	90	25	90	290	96	[45]
			85		100			Less than 300		
Mg-9Al alloy	Blended Mg with size 42 μm, irregular shape, and Al 17 μm, spherical shape	Laser-powder bed fusion	10	30–75	10	50	80	250	74.5	[46]
			15		20			187	78	
			15		40			94	86.1	
			20		40			125	82	
Mg-9Al alloy	Blended Mg with size 24 μm, spherical shape, and Al 28 μm, spherical shape	Laser-powder bed fusion	70	80	500	30	30	156	95.7	[47]
					750			104	88	
					1000			78	83	
					1250			63	81	
AZ61 alloy	Pre-alloyed with powder size 48 μm, spherical shape	Laser-powder bed fusion	145	75	300	45	65	210	Less than 99	[48]
					350			181		
					400			158		
					450			141		
					400		85	157	99.1	
					400		110	95	98.1	
AZ61 alloy	Powder size with 70 μm, spherical shape	Laser-powder bed fusion	65	155	400	55	55	6000	77	[49]
			75					7000	89	
			85					8000	99	
			95					9000	95	
AZ91 alloy	Powder size with 59 μm, spherical shape	Laser-powder bed fusion	210		333	45	95	168	99.57	[50]
AZ91 alloy	Powder size with 53–75 μm, spherical shape	Laser-powder bed fusion	125	85	10	355	510	70	96.62	[51]
AZ91 alloy	Powder size with 25–63 μm, spherical shape	Laser-powder bed fusion	110	95	800	35	45	105	Less than 99	[52]
AZ91 alloy	Powder size with 30 μm, spherical shape	Laser-powder bed fusion	45	–	200	35	35	279	98	[53]
AZ91/SiC composite	Powder size with 50 nm, SiC particles								98.2	
WE43 alloy	Powder size with 25 μm, spherical shape	Laser-powder bed fusion	125	95	960	35	45	105	98.5	[33]
	Powder size with 30 μm, spherical shape		145		1200			105	99.1	
	Powder size with 63 μm, spherical shape		310		1200			209	99.4	
WE43 alloy	Powder size with 25–63 μm, spherical shape	Laser-powder bed fusion	205	95	700	35	45	239	99.78	[54]
WE43 alloy	Powder size with 25–63 μm, spherical shape	Laser-powder bed fusion	205	75	1100	45	135	38	99.6	[55]
WE43 alloy	Powder size with 25–63 μm, spherical shape	Laser-powder bed fusion	205	130	700	35	45	239	99.89	[56]
WE43 alloy	Powder size with 25–63 μm, spherical shape	Laser-powder bed fusion	200	110	800	35	210	42	99.75	[57]
			200		800		245	35	98.4	
			200		1200		210	29	96.6	
			200		1200		210	20	87.7	
G10K alloy	Powder size with 63 μm, spherical shape	Laser-powder bed fusion	85	–	200	35	90	135	99.3	[58]
GZ112K alloy	Powder size with 31–44 μm, spherical shape	Laser-powder bed fusion	85	110	100	35	90	268	98.9	[59]
					300			90	99.8	
					500			54	99.4	
					700			39	99.5	
					1000			28	96.4	
					1500			19	71.9	
					500		45	106	99.4	
					500		145	37	96.4	
GZ151K alloy	Powder size with 25–65 μm, spherical shape	Laser-powder bed fusion	210	–	700	35	75	138	98	[60]
Mg-1Zn alloy	Blended Mg-5.5Zn with Powder size of 36 μm involving Mg powder size 31 μm and Zn powder size 19 μm; spherical shape	Laser-powder bed fusion	185	140	700	25	75	184	99.1	[61]
Mg-2Zn alloy									98.5	
Mg-6Zn alloy									94.9	
Mg-12Zn alloy									99	
ZK60 alloy	Powder size with 30 μm, spherical shape	Laser-powder bed fusion	210	140	300	25	85	418	95	[62]
					500			255	94	
					700			180	89	
					900			140	85	

**Table 2:** Various properties of laser beam additively manufactured Mg-alloy.

Alloy	Input Energy Density (J/mm^3^)	Grain Size (μm)	Micro-Hardness (HV)	Yield Strength (MPa)	Ultimate Tensile Strength (MPa)	Elongation. (%)	Electrochemical Solution	Icorr (μA/cm^2^)	Mass Loss mm/Year	References
Mg (Pure)	97–88	1–5	–	–	–	–	Hank’s solution	75–180	5–33	[44]
Mg (Pure)	295	–	52.4	–	–	–	–	–	–	[45]
Mg-9Al	251	15–25	71	–	–	–		–	–	[46]
Mg-9Al	150	1.5–3.5		–	274	1.1	–	–	–	[47]
AZ61	140	1.5	–	220	275	3.5	–	–	–	[48]
	155	1.7		235	285	3.0				
	180	2.0		220	260	2.9				
	205	2.4		214	240	2.2				
AZ61	125	4.5	71	–	–	–	Simulated body fluid solution		2.8	[49]
	145	9	81						2.5	
	161	10	94						1.3	
	181	12	91						1.6	
AZ91	165–85	1.5–3	86–105	27	296	1.2				[50]
	83	2.9		237	254	1.8				
AZ91	68	1–11	114	–	–	–				[51]
AZ91	103	1–2	–	270	330	3.9				[52]
AZ91	280	3.5	–	310	350	1.1	–	–	–	[53]
AZ91-SiC	280	1.2	–	265	310	2.1	–	–	–	
AZ91–2Ca	–	–	–	240	335	3.3	–	–	–	[27]
WE43	125	35	–	–	–	–	NaCl (0.1 M)	5.1	6.1	[27]
	150	28						5.0	–	
	300	19						4.6		
WE43	240	1.5	–	300	310	12.1	–	–	–	[54]
WE43	40	1–4	–	215	255	2.8	–	–	–	[55]
WE43	240	20.5	–	–	–	–	–	–	–	[56]
WE43										[57]
G10K	135	28	81	187	230	2.3	–	–	–	[58]
GZ112K	90	1.8	–	330	335	4.2	–	–	–	[59]
GZ151K	140	2.1		350	370	3.2	–	–	–	[60]
Mg-1Zn	185	–	52		150	11.1	–	–	–	[61]
Mg-2Zn			45		75	2.4				
Mg-6Zn			66		55	1.4				
Mg-12Zn			84		80	3.3				
ZK30	2000	–	81	–	–	–	Simulated body fluid solution	17.8	1.20	[27]
ZK30-Cu			99					47.8	2.25	
Pure Mg	–	–	–	–	–	–	NaCl (3 wt.%)	999	144	[27]

**Table 3:** Effects of heat treatment on the mechanical characteristics of Aluminum alloys.

S.No	Aluminum Alloys	Condition of Heat Treatment	Yield Strength (MPa)	Ultimate Tensile Strength (MPa)	Elongation (%)	References
1	AlSi10Mg	(i) As-built (ii) 540 °C–1 h,180 °C–2 h	(i) 264 (ii) 277	(i) 451 (ii) 331	(i) 8 and 6 (ii) 5 and 8	[237]
2	AlSi10Mg	(i) As-built (ii) 550 °C–1 h,180 °C–2 h	(i) 225 (ii) 270	(i) 429 (ii) 321	(i) 4 (ii) 9	[242]
3	AlSi7Mg	(i) As-built (ii) 540 °C–1 h,160 °C–4 h	(i) 257 (ii) 256	(i) 398 (ii) 306	(i) 7 and 6 (ii) 4 and 7	[243]
4	AlSi10Mg	(i) As-built (ii) 540 °C–1 h,180 °C–8 h	(i) 275 (ii) 236	(i) 406 (ii) 288	(i) 3 and 8 (ii) 9 and 3	[241]
5	AlSi10Mg	(i) As built (ii) 535 °C–1 h,190 °C–10 h	(i) 270 (ii) 164	(i) 446 (ii) 214	(i) 8 and 1 (ii) 11	[244]
6	AlSi10Mg	(i) As built (ii) 540 °C–1 h,160 °C–6 h	(i) 360 (ii) 290	(i) 307 (ii) 267	(i) 1 and 7 (ii) 2 and 5	[245]
7	AlSi7Mg	(i) As-built (ii) 160 °C–8 h	(i) 225 (ii) 280	(i) 375 (ii) 400	(i) 7 and 5 (ii) 5 and 5	[246]
8	AlSi10Mg	(i) As-built (ii) 200 °C–2 h	(i) 263 (ii) 298	(i) 473 (ii) 479	(i) 7 and 8 (ii) 5 and 6	[247]
9	AlSi10Mg	(i) As-built (ii) 175 °C–1 h	(i) 267 (ii) 310	(i) 391 (ii) 440	(i) 5 and 6 (ii) 4 and 4	[248]
10	AlSi10Mg	(i) As-built (ii) 160 °C–5 h	(i) 255 (ii) 268	(i) 377 (ii) 342	(i) 2 and 2 (ii) 0 and 9	[249]
11	AlSi10Mg	(i) As-built (ii) 160 °C–4 h	(i) 257 (ii) 309	(i) 398 (ii) 411	(i) 7 and 6 (ii) 4 and 8	[250]

## Data Availability

Not applicable.
